# GPR15-mediated T cell recruitment during acute viral myocarditis facilitated virus elimination and improved outcome

**DOI:** 10.1038/s44161-023-00401-z

**Published:** 2023-12-27

**Authors:** Bastian Stoffers, Hanna Wolf, Lucas Bacmeister, Svenja Kupsch, Tamara Vico, Timoteo Marchini, Maria A. Brehm, Isabell Yan, P. Moritz Becher, Armin Ardeshirdavani, Felicitas Escher, Sangwon V. Kim, Karin Klingel, Paulus Kirchhof, Stefan Blankenberg, Tanja Zeller, Dennis Wolf, Ingo Hilgendorf, Dirk Westermann, Diana Lindner

**Affiliations:** 1 Department of Cardiology and Angiology, University Heart Center Freiburg-Bad Krozingen, Faculty of Medicine, University of Freiburg, Freiburg, Germany; 2 DZHK (German Centre for Cardiovascular Research), partner site Hamburg/Kiel/Lübeck, Hamburg, Germany; 3 Department Digital Health Sciences and Biomedicine, School of Life Sciences, University of Siegen, Siegen, Germany; 4 Department of Cardiology, University Heart & Vascular Centre Hamburg, University Medical Centre Hamburg-Eppendorf, Hamburg, Germany; 5 DZHK (German Centre for Cardiovascular Research), partner site Berlin, Berlin, Germany; 6 Institute for Cardiac Diagnostics and Therapy, Berlin, Germany; 7 Deutsches Herzzentrum der Charité, Department of Cardiology, Angiology and Intensive Care Medicine, Campus Virchow Klinikum, Berlin, Germany; 8 Department of Microbiology and Immunology, Sidney Kimmel Medical College, Thomas Jefferson University, Philadelphia, PA USA; 9 Cardiopathology, Institute of Pathology and Neuropathology, University Hospital Tübingen, Tübingen, Germany

**Keywords:** Cardiovascular diseases, Infectious diseases, Inflammation

## Abstract

Viral myocarditis is characterized by infiltration of mononuclear cells essential for virus elimination. GPR15 has been identified as a homing receptor for regulatory T cells in inflammatory intestine diseases, but its role in inflammatory heart diseases is still elusive. Here we show that GPR15 deficiency impairs coxsackievirus B3 elimination, leading to adverse cardiac remodeling and dysfunction. Delayed recruitment of regulatory T cells in GPR15-deficient mice was accompanied by prolonged persistence of cytotoxic and regulatory T cells. In addition, RNA sequencing revealed prolonged inflammatory response and altered chemotaxis in knockout mice. In line, we identified GPR15 and its ligand GPR15L as an important chemokine receptor–ligand pair for the recruitment of regulatory and cytotoxic T cells. In summary, the insufficient virus elimination might be caused by a delayed recruitment of T cells as well as delayed interferon-γ expression, resulting in a prolonged inflammatory response and an adverse outcome in GPR15-deficient mice.

## Abstract (web summary)

Stoffers, Wolf et al. report that, in virus-mediated myocarditis, GPR15 deficiency delays the recruitment of T cells into cardiac tissue, which impairs viral elimination and leads to delayed but also prolonged inflammation and, thus, adverse cardiac outcomes.

## Main

Myocarditis is an inflammatory disease of the myocardium characterized by mononuclear cell infiltration^1^. It is predominantly caused by infectious agents, such as the cardiotropic enterovirus coxsackievirus B3 (CVB3)^2^. Especially in young adults, myocarditis is a major source of sudden cardiac arrest^3–5^. However, its clinical course has a broad spectrum of outcomes, ranging from mild symptoms and complete recovery to cardiac dysfunction and dilated cardiomyopathy (DCM)^2,6^. In more than 65% of patients with DCM of unknown etiology, viral genomes were detected in the myocardium^6^. DCM after acute viral myocarditis arises due to sustained inflammation predominantly caused by insufficient virus clearance and subsequent virus persistence in the cardiac tissue^3,7,8^. Thus, virus elimination and dampening cardiac inflammation are eminent steps toward complete recovery for patients suffering from myocarditis. As therapeutic strategies for viral myocarditis are limited, regulators orchestrating virus elimination and cardiac inflammation may display innovative targets for future treatment.

Chemoattractant G-protein-coupled receptors (GPCRs) are critical regulators in recruiting lymphocyte subsets from blood and secondary lymphoid organs to peripheral tissues, such as the heart. Furthermore, they are involved in the migration of these immune cells between and within these organs and tissues in both homeostatic and inflammatory states^9,10^.

Based on sequence similarities, G-protein-coupled receptor 15 (GPR15) was discovered in 1996 as a chemokine receptor^11,12^. Functionally, it has been identified as a T cell homing receptor in the context of inflammatory intestine and skin diseases^13–17^. Because GPR15 controls the specific homing of anti-inflammatory FOXP3^+^ regulatory T (T_reg_) cells to the large intestine in mice, GPR15-deficient mice developed more severe inflammation during colitis^15^. Furthermore, GPR15 is important not only for recruiting T_reg_ cells to the mouse colon but also for effector T cell recruitment in both homeostatic and inflammatory conditions^13^. Two ligands are known for GPR15: a subunit of the membrane protein thrombomodulin and the chemokine-like ligand GPR15L^18–20^.

GPR15-mediated recruitment of T_reg_ cells to the gut is essential for dampening inflammation during colitis in mice^15^, but the role of GPR15 in inflammatory heart diseases is still elusive. In this study, we used the murine model of viral myocarditis of C57Bl/6J (B6) mice, using cardiotropic CVB3 (ref. ^21^). The B6 mouse strain has a low susceptibility to CVB3 infection, which predominantly results in virus elimination after acute myocarditis^22,23^. In B6 mice, CVB3-induced myocarditis typically takes place in a three-phased manner^1,24^. During the first days (days 2–4) post infection (p.i.), CVB3 itself exerts direct cardiotoxic damage, followed by a highly inflammatory phase of immune cell infiltration (days 4–14). In this latter phase, host immunity mostly eliminates the virus from cardiac tissue^23^. The subsequent stage is characterized by the recovery and reversion of cardiac remodeling. Employing GPR15-deficient mice, we investigated the subacute phase (16 days p.i.) and found that GPR15 deficiency affected the outcome of CVB3-induced myocarditis. To elucidate the patho-mechanistical function of GPR15 during myocarditis, we thoroughly investigated its acute phase (5 days, 6 days and 7 days p.i.). Thereby, we focused on the following questions. Does GPR15 deficiency (1) lead to differences in immune cell infiltrate composition or cytokine expression in the heart during the acute phase of myocarditis; (2) affect the efficient elimination of CVB3 in the heart, thereby altering the outcome; and (3) have an impact on chemotactic migration, adhesion or functionality of T cells?

## Results

### Poor recovery from myocarditis in GPR15-deficient mice

**Fig. 1:**
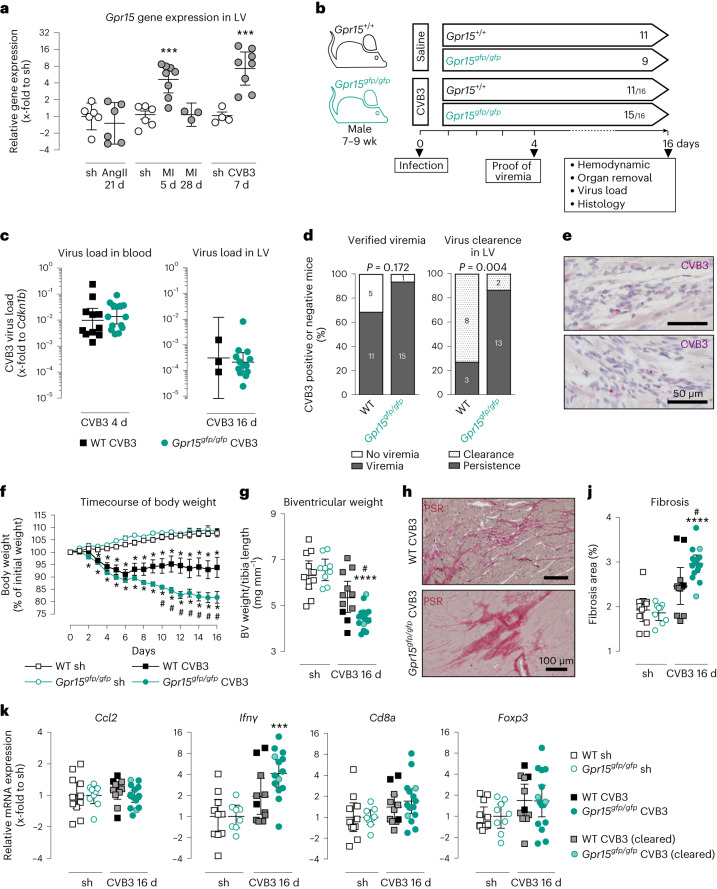
Impact of GPR15 deficiency during the subacute phase of CVB3-induced myocarditis 16 days p.i. **a**, *Gpr15* expression in LV tissue of WT mice with angiotensin II-induced hypertension (AngII), myocardial infarction (MI) and myocarditis (CVB3). Ct values were normalized to the mean of *18S* and *Cdkn1b* and to the corresponding sham controls (ΔΔCt). Unpaired two-tailed *t*-test (AngII and CVB3) or ordinary one-way ANOVA with Holm–Sidak correction (MI). *n* numbers are represented by data points. **b**, Study design: CVB3-injected mice, which revealed viral RNA neither in blood (4 days) nor in LV (16 days), were excluded from subsequent analyses. Within the arrow: *n* numbers per group, with CVB3^+^ mice and the original *n* number separated by a backslash. **c**, Virus load in blood and LV tissue. Ct values were normalized to *Cdkn1b* (ΔCt). Unpaired two-tailed *t*-test. *n* numbers according to **b**. **d**, Number of mice with verified viremia (4 days) or virus persistence in LV (16 days). *n* numbers according to **b** are plotted as stacked bar charts. Two-tailed Fisher’s exact test was performed on the underlying contingency table. **e**, In situ hybridization visualized a representative region with viral RNA (purple) in LV tissue from two CVB3^+^ *Gpr15*^*gfp/gfp*^ mice with co-stained nuclei (blue). Two technical replicates. **f**, Body weight in relation to individual initial weight (mean ± s.e.m.). Two-tailed multiple *t*-tests with Holm–Sidak correction. **g**, Ratio of biventricular weight to tibia length (mean ± 95% CI). Unpaired two-tailed *t*-test with Bonferroni correction. **h**, Representative histological PSR staining of biventricular tissue. **j**, Quantification of fibrotic areas (mean ± 95% CI). Unpaired two-tailed *t*-test with Bonferroni correction. **k**, *Ccl2*, *Ifnγ*, *Cd8a* and *Foxp3* expression in LV. Symbols filled in gray and light green indicate mice that cleared virus from LV tissue. Ct values were normalized to the mean of *18S* and *Cdkn1b* and corresponding sham controls (ΔΔCt). Unpaired two-tailed *t*-test with Bonferroni correction. **a**,**d**,**k**, Gene expression data were plotted as 2^−ΔCt^ or 2^−ΔΔCt^ (geo-mean ± 95% CI). Significant, compared * to sham of the same genotype, # between similarly treated groups of different genotypes. (*, ***, ****; *P* < 0.05, 0.001, 0.0001). d, days; sh, sham; wk, weeks. Source data

We examined the regulation of GPR15 in various heart failure entities (Fig. 1a). During the acute phase of CVB3-mediated myocarditis, *Grp15* expression was 7.3-fold increased, which was the strongest increase in the heart failure models investigated. Therefore, we aimed to examine the role of GPR15 during viral myocarditis.

To explore whether GPR15 has an impact on progression of or recovery from viral myocarditis, we first investigated the subacute phase 16 days p.i. Mice with GPR15 deficiency were employed as depicted in Fig. 1b. To prove infection, we verified viremia in small blood samples at day 4 p.i. As depicted in Fig. 1b,d, mice without verified viremia were excluded from subsequent analyses.

Albeit virus load in blood 4 days p.i. was not different between both genotypes (Fig. 1c), significantly more GPR15-deficient mice had persisting virus in left ventricular (LV) tissue at 16 days p.i. Remarkably, 73% of CVB3-infected WT mice (eight of 11) but only 13% of CVB3-infected GPR15-deficient mice (two of 15) cleared the virus from the heart after 16 days (Fig. 1d). In situ hybridization on LV tissue sections revealed that viral RNA was hardly detectable in the subacute phase. As exemplarily shown in Fig. 1e, viral RNA was mainly localized in non-myocyte cells. Despite the inefficient virus elimination from LV tissue, gene expression of chemokine (*Ccl2*) or T cell markers (*Cd8a*, cytotoxic T (T_C_) cells / *Foxp3*, T_reg_ cells) was not different compared to either infected wild-type (WT) mice or corresponding sham controls (Fig. 1k). Furthermore, CVB3-infected mice, with or without effective virus clearance, revealed similar gene expression of inflammatory markers compared to the respective sham group. However, the significantly increased gene expression of anti-viral *Ifnγ* in infected GPR15-deficient mice is in line with the detected virus persistence. Decreasing body weight was observed in both infected groups until 6 days p.i. Although WT mice stabilized their body weight afterwards, the weight loss further proceeded in GPR15-deficient mice, construed as a sign of aggravating disease severity (Fig. 1f). This divergent timecourse of body weight started on day 7. In line with this finding, GPR15-deficient mice revealed significantly lower heart weights compared to WT mice at 16 days p.i. (Fig. 1g). This was accompanied by larger lesions, presumably owing to cardiomyocyte death, resulting in more reparative cardiac fibrosis in GPR15-deficient mice (Fig. 1h,j). Interestingly, two of the three WT mice without virus clearance had clearly larger fibrotic lesions than those that had cleared the virus from LV tissue.

Additionally, pressure–volume (PV) loops revealed cardiac dysfunction in both infected groups, indicating that they did not restore their cardiac function in the subacute phase of myocarditis (Table 1). However, GPR15-deficient mice showed a tendency to more impaired cardiac function. Analyzing parameters that specifically characterize systolic function, solely CVB3-infected GPR15-deficient mice showed significantly impaired cardiac function compared to their sham controls, whereas comparison of WT groups did not reach significance. This is displayed, for example, by impaired preload-independent and preload-dependent cardiac contractility (preload-recruitable stroke work (PRSW) −23%, *P* = 0.0256 and ΔP/Δt_max_ −29%, *P* = 0.0272) in GPR15-deficient mice but almost unaffected contractility in WT mice. Analysis of diastolic function revealed significantly slowed relaxation and increased myocardial stiffness (ΔP/Δt_min_ +27%, *P* = 0.0151 and Tau +21%, *P* = 0.0373) in GPR15-deficient mice, which is in line with increased cardiac fibrosis determined by histology (Fig. 1h,j). Additionally, comparing both infected groups normalized to their sham controls, significantly impaired parameters, such as preload-independent cardiac contractility (PRSW −19%, *P* = 0.0063), maximal pressure (P_max_ −11%, *P* = 0.0175) and relaxation (ΔP/Δt_min_ +24%, *P* = 0.0016), were determined in GPR15-deficient mice (Table 1). Even virus clearance and resolved cardiac inflammation, mainly observed in infected WT mice, did not result in preserved cardiac function but may prevent more severe cardiac dysfunction, as detected in GPR15-deficient mice with predominant virus persistence.

**Table 1:** Cardiac function in the subacute phase (16 days p.i.) of myocarditis characterized by PV loops

	Sham groups		CVB3 myocarditis groups							
	WT	*Gpr15*^*gfp/gfp*^	WT	vs. Sh		*Gpr15*^*gfp/gfp*^	vs. Sh		*Gpr15*^*gfp/gfp*^ vs. WT	
				Change (%)	*P* value^a^		Change (%)	*P* value^a^	Change (%)	*P* value^b^
Global function										
Heart rate (bpm)	571 ± 16	613 ± 13	553 ± 19	−3	1.0000	552 ± 19	−10	0.0544	−7	0.1514
Cardiac output (ml min^−1^)	18.3 ± 1	19.2 ± 1.8	14.3 ± 1.9	−22	0.3406	13.2 ± 1.3	−31	**0.0243**	−12	0.4487
Stroke volume (µl)	32 ± 1	31 ± 3	26 ± 3	−20	0.2492	24 ± 2	−23	0.0876	−4	0.7987
Stroke work (µl × mmHg)	2,699 ± 146	2,737 ± 270	2,046 ± 270	−24	0.2218	1,707 ± 172	−38	**0.006**	−18	0.2533
Systolic function										
Ejection fraction (%)	80 ± 7	74 ± 4	59 ± 6	−26	0.1042	59 ± 5	−21	0.1056	+6	0.6219
PRSW (mmHg)	75.2 ± 3.2	78.6 ± 6.4	71.6 ± 3.3	−5	0.9513	60.3 ± 3.3	−23	**0.0256**	−19	**0.0063**
P_max_ (mmHg)	101 ± 4	103 ± 3	93 ± 3	−8	0.2366	84 ± 3	−19	**0.0001**	−11	**0.0175**
ΔP/Δt_max_ (mmHg s^−1^)	10,786 ± 563	12,379 ± 1225	9,943 ± 1053	−8	1.0000	8,759 ± 726	−29	**0.0272**	−23	0.0642
Diastolic function										
P_ed_ (mmHg)	5 ± 1	3.9 ± 1	3.6 ± 0.7	−28	0.5618	3.6 ± 0.9	−7	1.0000	+29	0.4593
ΔP/Δt_min_ (mmHg s^−1^)	−9,001 ± 453	−10,246 ± 906	−8,684 ± 428	+4	1.0000	−7,531 ± 436	+27	**0.0151**	+24	**0.0016**
Tau (ms)	4.9 ± 0.1	4.5 ± 0.3	5.1 ± 0.3	+4	1.0000	5.4 ± 0.2	+21	**0.0373**	+16	0.0648

Values are given as mean ± s.e.m. ^a^Two-tailed *t*-test with Bonferroni correction. ^b^Comparison between infected genotypes was based on values normalized to the corresponding sham group. Unpaired two-tailed *t*-test. Due to normalization, only one comparison was made, and *P* value was, therefore, not corrected for multiple testing. *P* values indicting significance are highlighted in bold. WT sh/CVB3 (*n* = 6/11); *Gpr15*^*gfp/gfp*^ sh/CVB3 (*n* = 9/13).

P_max_, maximum pressure; ΔP/Δt_max_, maximal rate of rise of LV pressure; P_ed_, end-diastolic pressure; ΔP/Δt_min_, maximal rate of decrease of LV pressure; Sh, sham.

### Persistently high virus load in *Gpr15*^*gfp/gfp*^ mice

**Fig. 2:**
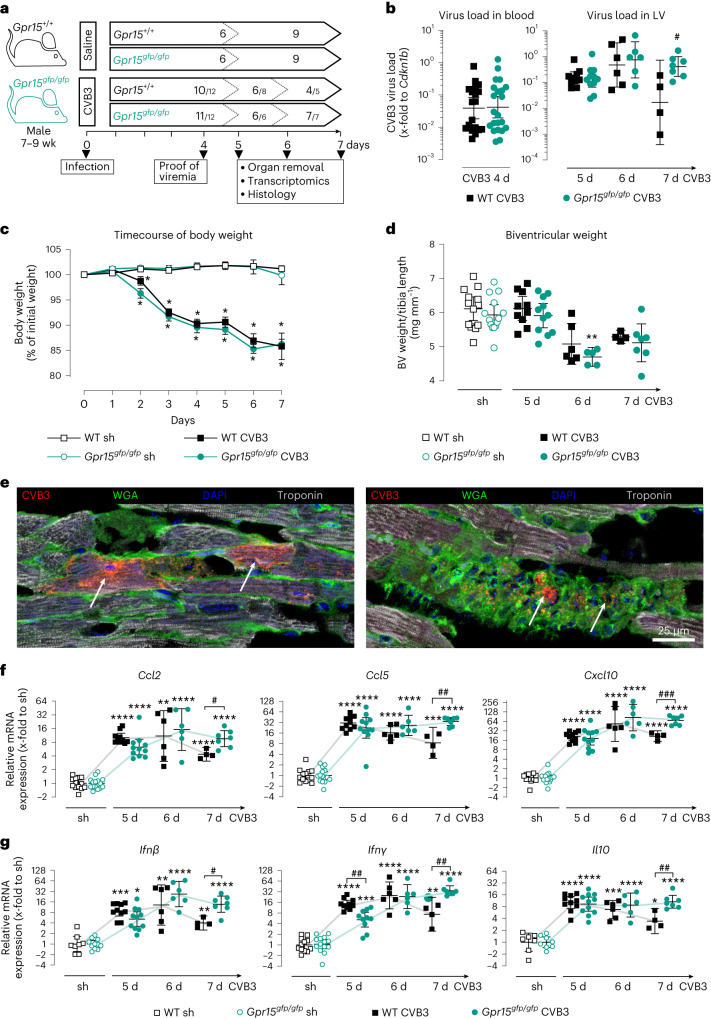
Impact of GPR15 deficiency during acute phase of CVB3-induced myocarditis 5 days, 6 days and 7 days p.i. **a**, Study design to investigate the impact of GPR15 in acute myocarditis. *n* numbers per group and timepoint are specified within the arrow. CVB3-injected mice, which revealed viral RNA neither in blood (4 days) nor in LV (5 days, 6 days or 7 days), were excluded from subsequent analyses. *n* numbers of CVB3^+^ mice included is given, with the original *n* number after the backslash. **b**, Virus load was determined in blood and LV tissue. Ct values were normalized to *Cdkn1b* (ΔCt). **c**, Body weight plotted as percentage to the individual initial weight (mean ± s.e.m.). Two-tailed multiple *t*-tests with Holm–Sidak correction. **d**, Biventricular weight normalized to tibia length (mean ± 95% CI). Unpaired two-tailed *t*-test with Bonferroni correction. **e**, In situ hybridization visualized viral RNA (red) in cardiac tissue from a CVB3-infected WT mouse co-stained with WGA (green) and troponin (white). Arrows point to two infected cardiomyocytes (left) or infected infiltrated cells (right). *n* = 2 mice in two independent experiments. **f**,**g**, Gene expression was determined in LV tissue of WT and GPR15-deficient mice (**f**) from selected chemokines (*Ccl2*, *Ccl5* and *Cxcl10*) (**g**), from selected anti-viral interferons (*Ifnβ* and *Ifnγ*) and from the anti-inflammatory interleukin *Il10*. Ct values were normalized to the mean of *18S* and *Cdkn1b* and the corresponding sham controls (ΔΔCt). **b**,**f**,**g**, Gene expression data were plotted as 2^−ΔCt^ or 2^−ΔΔCt^ (geo-mean ± 95% CI). Unless stated otherwise, significance was tested using an unpaired two-tailed *t*-test with Bonferroni correction. Significant, compared * to sham of the same genotype, # between similarly treated groups of different genotypes. (*, **, ***, ****; *P* < 0.05, 0.01, 0.001, 0.0001). d, days; sh, sham; wk, week. Source data

Because we hypothesized that the observed differences arise from the acute phase of myocarditis, we investigated CVB3-induced myocarditis in more detail on days 5, 6 and 7 p.i., as depicted in Fig. 2a. Virus load in blood at day 4 p.i. and virus load in LV tissue at 5 days and 6 days p.i. revealed no differences between genotypes (Fig. 2b). At day 7 p.i., however, the virus load in LV tissue of WT mice was significantly lower than in *Gpr15*^*gfp/gfp*^ mice, which remained at a high level. Timecourse of body weight showed significant weight loss for both infected groups but no differences between genotypes (Fig. 2c). Compared to sham controls, biventricular heart weight tended to be lower in CVB3-infected mice 6 days and 7 days p.i. in both genotypes (Fig. 2d) but reached significance only in *Gpr15*^*gfp/gfp*^ mice on day 6 p.i. With respect to the significantly lower biventricular weight in GPR15-deficient mice at 16 days p.i. (Fig. 1g), we suggest that a disease progression in GPR15-deficient mice becomes apparent at a later timepoint, which would be in line with the decrease in body weight after day 7. Viral RNA visualized by in situ hybridization was localized in cell infiltrates stained with wheat germ agglutinin (WGA) and frequently in cardiomyocytes identified by troponin staining (Fig. 2e), which was in contrast to the subacute phase.

### Prolonged cardiac inflammation in *Gpr15*^*gfp/gfp*^ mice

To investigate the innate and adaptive immune responses in the LV tissue after CVB3 infection, gene expression of chemotactic, anti-viral and anti-inflammatory cytokines was determined (Fig. 2f,g). Overall, these cytokines were significantly increased in the infected LV tissue at 5 days, 6 days and 7 days p.i. compared to respective sham groups, revealing strongly induced cytokine expression during acute myocarditis in both genotypes. Comparing the infected genotypes, no significant differences were observed at 6 days p.i. in contrast to 5 days and 7 days p.i. At 5 days p.i., GPR15-deficient mice showed significantly lower gene expression of *Ifnγ*. At 7 days p.i., however, infected GPR15-deficient mice exhibited significantly higher gene expression of *Ccl2*, *Ccl5*, *Cxcl10*, *Ifnβ*, *Ifnγ* and *Il10*. This might be caused by declined cytokine levels in infected WT mice but still increased cytokine levels in infected GPR15-deficient mice on day 7.

**Fig. 3:**
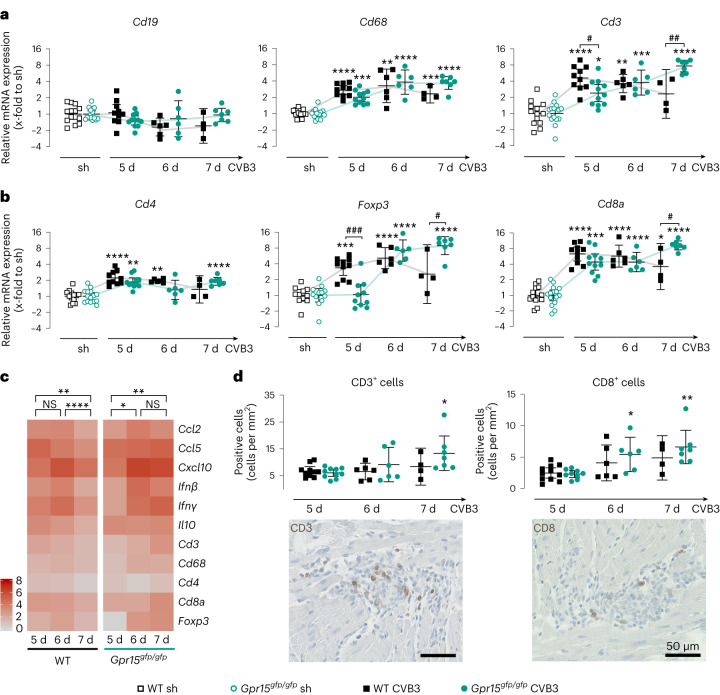
Analysis of immune cell infiltration in LV tissue during the acute phase 5 days, 6 days and 7 days after CVB3 infection. **a**,**b**, Gene expression was determined in LV tissue of WT and GPR15-deficient mice (*n* numbers stated in Fig. 2a) from specific immune cell markers for T cells (*Cd3*), B cells (*Cd19*) and macrophages (*Cd68*) (**a**) and markers for the T cell subpopulations T_H_ (*Cd4*), T_C_ (*Cd8*) and T_reg_ cells (*Foxp3*) (**b**). Ct values were normalized to the mean of *18S* and *Cdkn1b* and the corresponding sham controls (ΔΔCt). 2^−ΔΔCt^ values were plotted (geo-mean ± 95% CI). **c**, For all genes that were significantly regulated at any of the timepoints examined, the FC of gene expression compared to sham treatment is shown as a heat map. Significance was tested using a paired two-tailed *t*-test with Bonferroni correction. **d**, CD3^+^ and CD8^+^ cells were stained on tissue sections from CVB3-infected mice (*n* numbers stated in Fig. 2a) and then quantified and normalized to tissue area (mean ± 95% CI). Representative images show immune cell infiltrates in GPR15-deficient mice at 7 days p.i. Significance was tested using an unpaired two-tailed *t*-test with Bonferroni correction unless otherwise stated. Significant, compared * to sham of the same genotype, # between similarly treated groups of different genotypes. (*, **, ***, ****; *P* < 0.05, 0.01, 0.001, 0.0001). NS, not significant; sh, sham. Source data

During the immune response, various immune cells of the adaptive immune system, as well as monocytes and macrophages, are recruited to the infected LV tissue. Therefore, gene expression of specific immune cell markers was examined in LV tissue (Fig. 3a). Although the gene expression of the B cell marker *Cd19* was not altered compared to sham controls, the T cell marker *Cd3* and the macrophage marker *Cd68* were significantly increased in all infected groups. When comparing the infected genotypes, no significant differences were observed for *Cd68*, but *Cd3* was significantly different at 5 days and 7 days p.i. While *Cd3* expression at 5 days p.i. was significantly lower in LV tissue of GPR15-deficient mice, it was significantly higher at 7 days p.i. compared to WT mice.

**Extended Data Fig. 1:**
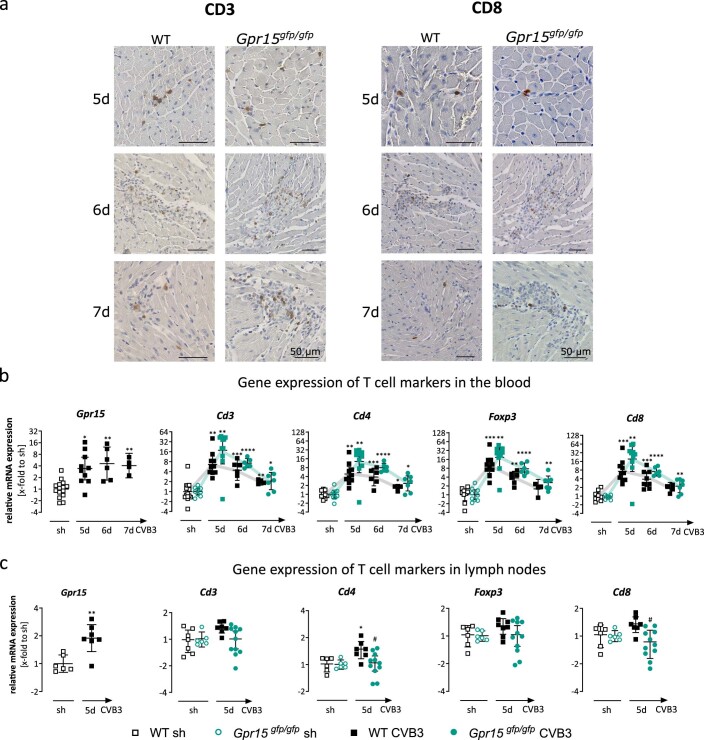
Analysis of immune cells in blood, lymph nodes and LV tissue sections. (a) Representative staining of CD3 (left panel) or CD8 (right panel) in immune cell infiltrates during the acute phase of myocarditis in heart tissue of infected WT and GPR15^−^deficient mice. Only infected mice were stained, n numbers are shown in Fig. 2a. (b) Gene expression of *Gpr15* was determined in blood of WT mice. N numbers stated in Fig. 2a. Gene expression of specific immune cell markers for T cells (*Cd3*) and T cell sub-populations T_H_ (*Cd4*), T_C_ (*Cd8*) and T_reg_ cells (*Foxp3*) was determined in blood of WT and GPR15-deficient mice. Ct-values were normalised to *18* *S* and the corresponding sham (sh) controls (ΔΔCt). 2^¬ ΔΔCt^ values were plotted (geo-mean ± 95% CI). Significance was tested using an unpaired two-tailed t-test with Bonferroni correction. (c) Gene expression of *Gpr15* was determined in lymph nodes of WT mice. N numbers stated in Fig. 2a. Gene expression of specific immune cell markers for T cells (*Cd3*) and T cell sub-populations T_H_ (*Cd4*), T_C_ (*Cd8*) and T_reg_ cells (*Foxp3*) was determined in lymph nodes of WT and GPR15-deficient mice. Ct-values were normalised to *Cdkn1b* and the corresponding sham (sh) controls (ΔΔCt). 2^¬ ΔΔCt^ values were plotted (geo-mean ± 95% CI). Unpaired two-tailed t-test with Bonferroni correction. Significant, compared * to sh of the same genotype, # between similarly treated groups of different genotypes. (*, **, ***, ****; *p* < 0.05, 0.01, 0.001, 0.0001) Source data

Because the expression of the T-cell-specific marker *Cd3* revealed significant differences between both genotypes on days 5 and 7 p.i., we subsequently examined markers for the T cell subpopulations T helper (T_H_, *Cd4*), T_C_ (*Cd8*) and T_reg_ cells (*Foxp3*). In general, CVB3 infection resulted in a slightly increased gene expression of *Cd4* but a strong increase of *Cd8* and *Foxp3* compared to sham controls (Fig. 3b). However, *Foxp3* was not increased in GPR15-deficient mice at 5 days p.i. When comparing the infected genotypes, significant differences were detected in the expression of *Foxp3* (on days 5 and 7 p.i.) and *Cd8* (on day 7 p.i.) but not in the expression of *Cd4*. Furthermore, none of these markers was differentially expressed on day 6 p.i. This indicates genotype-specific and temporal differences in the infiltration of T_reg_ and T_C_ cells. Although at day 5 p.i. *Foxp3* expression remained at basal levels in GPR15-deficient mice but was significantly increased in WT mice, on day 7 p.i. it was significantly higher in GPR15-deficient mice than in WT mice. This suggests delayed recruitment and prolonged persistence of T_reg_ cells. Furthermore, *Cd8* was not differentially expressed on day 5 and day 6, but it was significantly lower in WT mice at 7 days p.i. than in GPR15-deficient mice. The latter finding indicates prolonged persistence of T_C_ cells in the infected LV tissue of GPR15-deficent mice. We could strengthen the gene expression data by staining T cell subpopulations on LV tissue sections. Although no significant differences were detected between the two genotypes, significantly more CD3^+^ and CD8^+^ cells were detected in the infected GPR15-deficient mice at day 7 and at days 6 and 7, respectively. Representative images of immune cell infiltrates are shown (Fig. 3d and Extended Data Fig. 1a).

Figure 3c summarizes the results of the gene expression measurements in terms of their temporal course as a heat map for each genotype individually. In WT mice, gene expression of cytokines and immune cell markers increased as early as day 5, remained elevated on day 6 and decreased significantly on day 7. In GPR15-deficient mice, the gene expression significantly increased from day 5 to day 6 p.i. and remained elevated until 7 days p.i. This summary suggests, again, a delayed and prolonged cardiac inflammation during acute myocarditis in GPR15-deficient mice.

In addition, we measured gene expression of the same T cell markers in blood and lymph nodes (Extended Data Fig. 1b,c). Five days p.i., the expression of all investigated markers (*Cd3*, *Cd4*, *Foxp3* and *Cd8*) were significantly increased in blood of CVB3-infected mice compared to sham controls and tended to be even higher in blood of infected GPR15-deficient mice. In contrast to blood, in lymph nodes, the expression of *Cd4* and *Cd8* was significantly lower at day 5, which was in line with the observations in LV tissue. This opposite effect implies that the T cells remain in the circulation due to delayed tissue invasion.

### More upregulated differentially expressed genes in *Gpr15*^*gfp/gfp*^ mice at 7 days p.i

We aimed to identify the patho-mechanisms behind the adverse outcome observed at day 16 p.i. Given the decreasing body weight, higher virus load and more severe cardiac inflammation in GPR15-deficient mice, we assumed that the divergent disease progression might start at day 7. Thus, we next investigated this turning point by whole transcriptome analysis to determine the differences in gene expression between infected WT and infected GPR15-deficient mice from days 6 and 7. Therefore, 3′ mRNA sequencing was performed on a set of LV tissue samples (six sham controls and 3–6 infected mice per genotype). RNA sequencing revealed more than 30,000 annotated genes whose expression was subsequently compared between different groups.

**Fig. 4:**
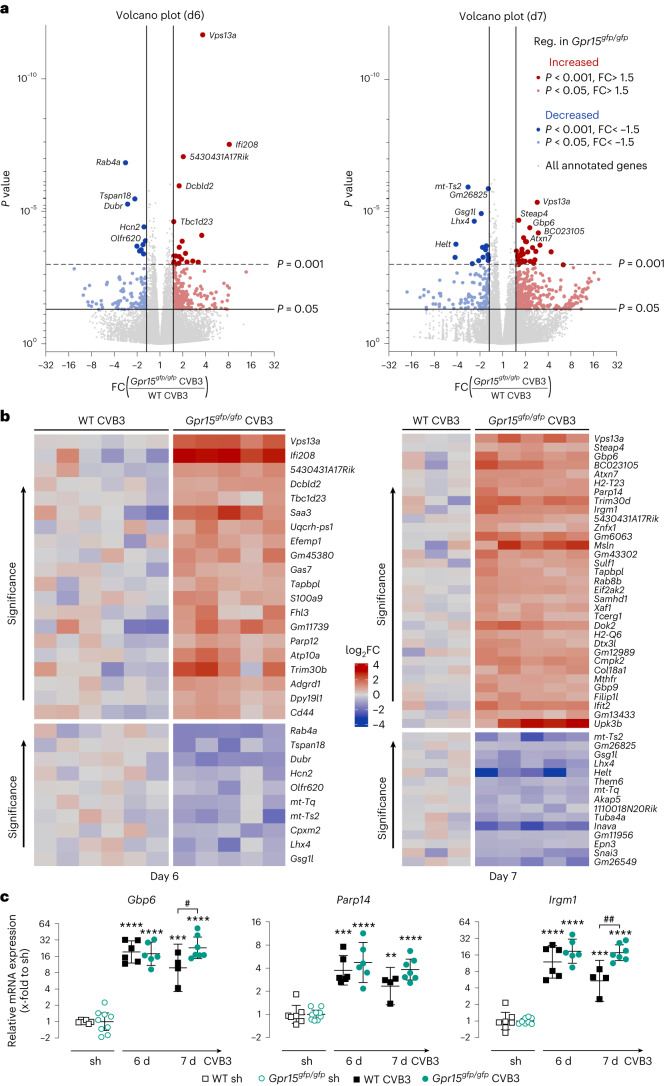
Identification of DEGs comparing cardiac tissue samples from *Gpr15*^*gfp/gfp*^ and WT mice at 6 days and 7 days after CVB3 infection. **a**, Transcriptome analysis was performed by bulk RNA sequencing of LV tissue from CVB3-infected *Gpr15*^*gfp/gfp*^ mice compared to infected WT mice at 6 days (left) and 7 days (right) p.i. FC on *x* axis and *P* value on *y* axis are displayed in the volcano plot. Genes that reveal an FC of at least ±1.5 and *P* < 0.05 are highlighted. DEGs downregulated in GPR15-deficient mice are shown in blue (*P* < 0.05, light blue; *P* < 0.001, dark blue), whereas upregulated DEGs are shown in red (*P* < 0.05, light red; *P* < 0.001, dark red). The five most significant upregulated and downregulated DEGs are labeled. DEGs were calculated with DESeq2 using the Wald test. **b**, Heat map of all top DEGs (FC ± 1.5 and *P* < 0.001) on day 6 (left) and day 7 (right) p.i. Gene expression is normalized to the mean of the respective infected WT group and plotted as FC (log_2_) for each sample separately. DEGs were calculated with DESeq2 using the Wald test. **c**, To validate sequencing data, gene expression of three top DEGs on day 7 (*Gbp6*, *Parp14* and *Irgm1*) was determined in all mice (*n* numbers stated in Fig. 2a). Ct values were normalized to the mean of *18S* and *Cdkn1b* and to the corresponding sham controls (ΔΔCt). Unpaired two-tailed *t*-test with Bonferroni correction. 2^−ΔΔCT^ values were plotted (geo-mean ± 95% CI). Significant, compared * to sham of the same genotype, # between similarly treated groups of different genotypes. (*, **, ***, ****; *P* < 0.05, 0.01, 0.001, 0.0001). d, days; Reg., regulated; sh, sham. Source data

**Extended Data Fig. 2:**
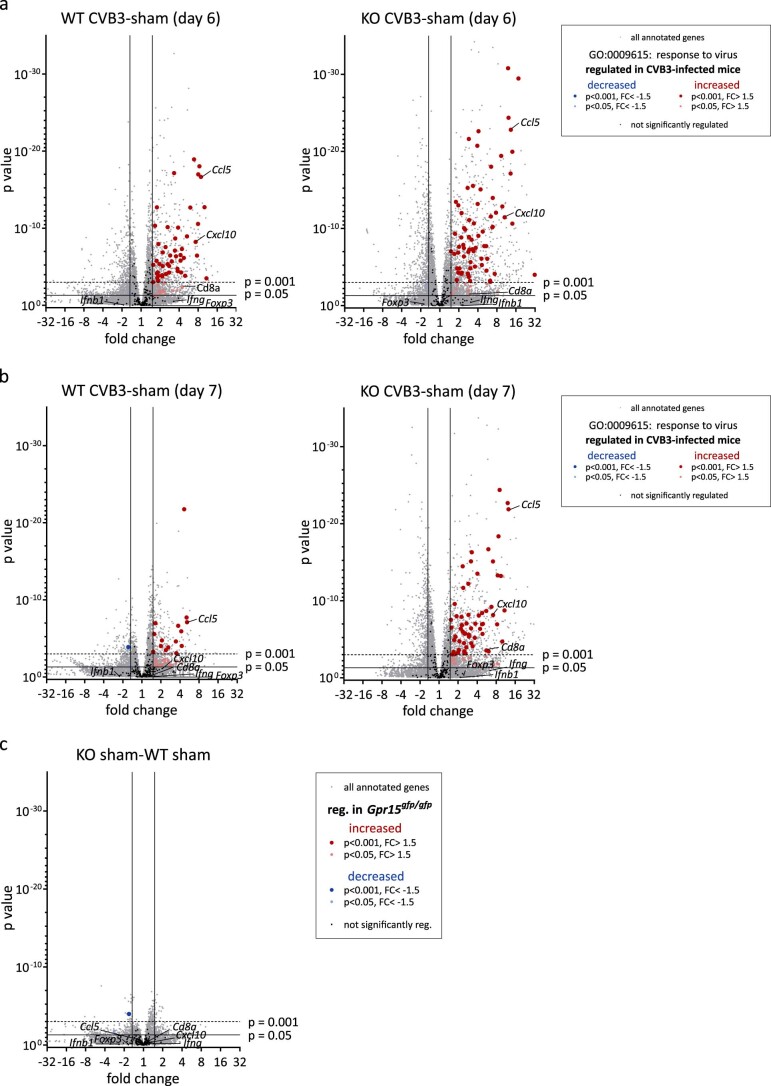
Volcano plots to visualise DEGs assigned to the GO term GO:0006915 “response to virus”. (a) Myocarditis – 6 days: CVB3-infected WT (left) and *Gpr15*^*gfp/gfp*^ (right) mice compared to their sham (sh) control 6 days p.i. (b) Myocarditis – 7 days: CVB3-infected WT (left) and *Gpr15*^*gfp/gfp*^ (right) mice compared to their sh control 7 days p.i. (c) Comparison of sh WT and sh *Gpr15*^*gfp/gfp*^ mice. Transcriptome analysis was performed by MACE-RNA seq of LV tissue. Fold change (FC) and *p*-value are displayed in the Volcano plot to visualise significant differences in gene expression. Genes assigned to the GO term GO:0006915 “response to virus” were highlighted as follows: (i) Not significantly regulated (black), (ii) significantly upregulated (FC > 1.5 & *p*-value < 0.05 (light red), *p*-value < 0.001 (dark red)) or (iii) significantly downregulated (FC < -1.5 & *p*-value < 0.05 (light blue), *p*-value < 0.001 (dark blue)). Genes labelled with their symbol are part of this GO term and were quantified by TaqMan analyses and plotted in Fig. 3. Genes not assigned to the GO term GO:0006915 are displayed in grey. DEGs were calculated with DESeq2 using Wald test. Source data

The 242 genes assigned to the Gene Ontology (GO) term GO:0006915 ‘response to virus’ are highlighted in the volcano plot in Extended Data Fig. 2. At 6 days p.i., approximately 30% of those genes were significantly increased in both genotypes, confirming the response to virus infection. At 7 days p.i., however, the number of significantly increased genes was diminished in WT mice to 16% but remained similar in GPR15-deficient mice. Lastly, both sham groups were compared, and only two slightly downregulated genes of that GO term were found in *Gpr15*^*gfp/gfp*^ mice. With respect to the TaqMan measurements in Figs. 3 and 4, we labeled the genes assigned to the highlighted GO term additionally with their gene symbol (*Ccl5*, *Cxcl10*, *Cd8a*, *Ifnβ*, *Ifnγ* and *Foxp3*). This demonstrates that the genes in the volcano plots revealed similar expression, as measured by TaqMan.

The comparison of infected genotypes is depicted as volcano plots in Fig. 4a for 6 days p.i. (left) and 7 days p.i. (right). Differentially expressed genes (DEGs) with a *P* value lower than 0.05 and a fold change (FC) higher or lower than 1.5 or −1.5 are highlighted. Comparing both volcano plots revealed more DEGs on day 7 (Fig. 4a, right). Although at 6 days p.i., 372 DEGs were detected (250 upregulated (red) and 122 downregulated (blue) in *Gpr15*^*gfp/gfp*^ mice), 540 DEGs were identified (404 upregulated (red) and 136 downregulated (blue)) at 7 days p.i. The DEGs were further restricted by *P* < 0.001, resulting in top DEGs. The 30 top DEGs for 6 days p.i. and the 48 top DEGs for 7 days p.i are displayed as a heat map in Fig. 4b.

To confirm the RNA sequencing data, three of the top DEGs from day 7 were quantified, including all mice, using TaqMan. Therefore, we selected genes related to immune response. As shown in Fig. 4c, the gene expression of *Gbp6*, *Parp14* and *Irgm1* was similar between both infected genotypes at 6 days p.i. and revealed higher expression in GPR15-deficient mice at 7 days p.i.

### Chemotaxis and T-cell-related GO terms are enriched at day 7

**Fig. 5:**
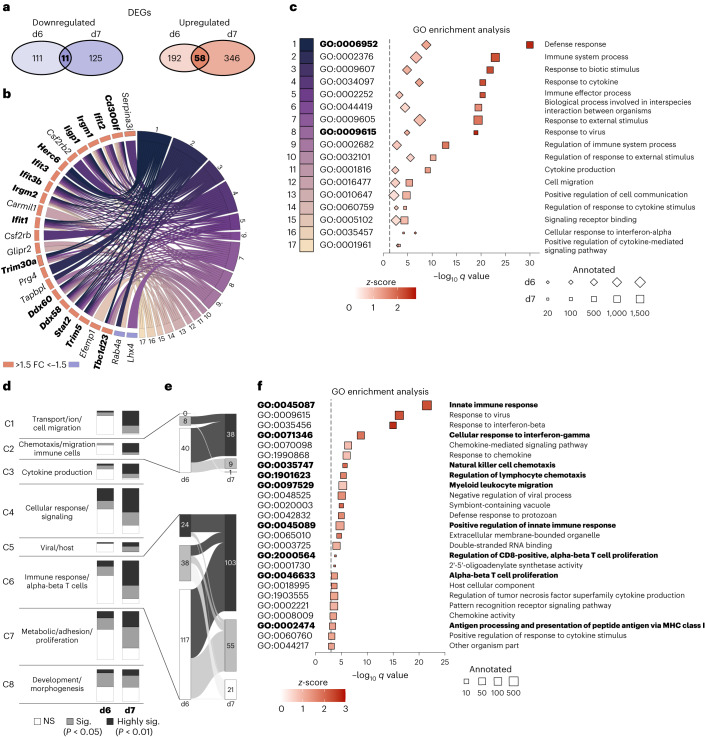
Upregulated GO terms related to T-cell-mediated immune response and chemotaxis in infected *Gpr15*^*gfp/gfp*^ mice on day 7 p.i. **a**, Venn diagram visualizes the overlap of upregulated and downregulated DEGs between the infected genotypes on both days p.i. Sixty-nine DEGs—58 upregulated and 11 downregulated—showed similar regulation on days 6 and 7 in GPR15-deficient mice. **b**, GO terms with at least three of the 69 DEGs were selected. Only GO terms with fewer than 2,000 genes and *P* < 0.01 were considered, further narrowed down by REVIGO and presented in the GO chord graph. The genes on the left side are connected by lines to the appertaining GO term on the right side. DEGs assigned to ‘response to virus’ and ‘defense response’ are highlighted in bold. **c**, GO terms from the GO chord (**b**) are depicted as a dot plot for days 6 and 7. GO terms are sorted according to the *q* value on day 7. **d**, Based on the differential expression between both infected groups, 957 GO terms of the domain BP were significantly regulated on day 6 and/or day 7. Using semantic similarity, GO terms were grouped into eight clusters. Within one cluster, the number of GO terms based on their *P* value is displayed as stacked bar charts to compare the distribution of significantly regulated GO terms between days 6 and 7. **e**, The number of GO terms of clusters 2 and 6 are shown in detail using alluvial plots. This visualizes changes of significantly regulated GO terms from day 6 to day 7. **f**, Dot plot of selected GO terms of all three domains between both infected groups on day 7. Selection is based on gene ratio (DEGs/annotated genes) greater than 0.1 and *q* < 0.001. Redundant and closely related GO terms were removed by REVIGO. GO terms assigned to clusters 2 and 6 are highlighted in bold. **c**,**f**, Size of the squares represents the number of annotated genes, and color represents the *z*-score. Red *z*-scores indicate more upregulated than downregulated DEGs in GPR15-deficient mice. Terms are sorted according to the –log_10_ of *q* value, which is displayed on the *x* axis. **b**–**f**, Significance of GO term enrichment was calculated by Fisher’s exact test based on DEGs (*P* < 0.05 and FC > +1.5 or FC < −1.5). Adjusted *P* values (*q* values) of GO terms were determined using the Benjamini–Hochberg correction. d, days; NS, not significant. Source data

**Extended Data Fig. 3:**
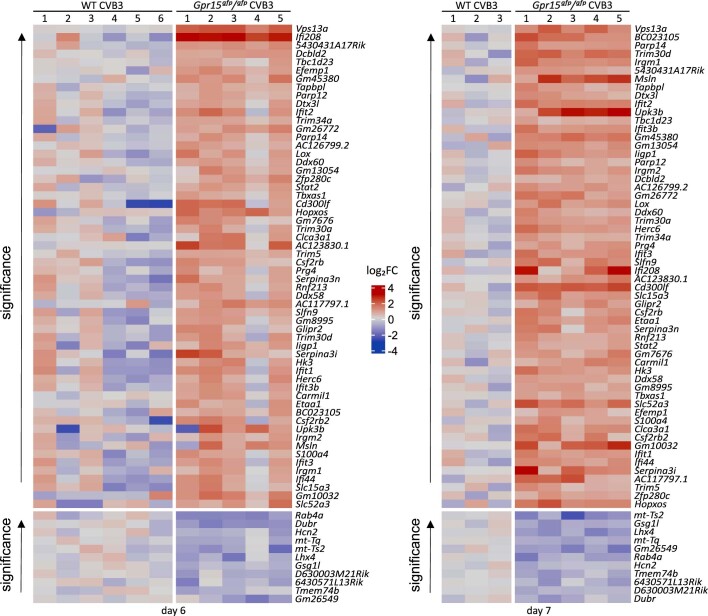
69 similar regulated DEGs found by comparing DEGs from day 6 and day 7. Heat maps of the 69 DEGs (58 up- and 11 downregulated in GPR15-deficient mice, FC + /-1.5 and a *p*-value < 0.001) that were similar regulated on day 6 (left) and 7 (right) p.i. Gene expression is normalized to the mean of the respective CVB3-infected WT group and plotted as fold change (FC, log_2_) for each sample separately. DEGs were calculated with DESeq2 using Wald test. Source data

To go beyond single genes, RNA sequencing data were further processed using GO enrichment analyses. First, we focused on general differences between the infected genotypes on both days. Comparing the DEGs from days 6 and 7, we identified 69 DEGs that were similarly regulated on both days (Fig. 5a). Although 11 DEGs were downregulated, 58 were upregulated in GPR15-deficient mice (Fig. 5a and Extended Data Fig. 3). Based on the overlapping DEGs, we identified GO terms including at least three of these 69 similarly regulated DEGs. GO terms with more than 2,000 annotated genes and *P* > 0.01 were excluded, and closely related or redundant GO terms were removed using REVIGO^25^. The remaining 17 terms with the assigned DEGs are depicted in the GO chord graph (Fig. 5b). GPR15-deficent mice revealed upregulated DEGs that were assigned to ‘response to virus’ and ‘defense response’ (highlighted in bold). The 17 GO terms are plotted as a dot plot to visualize their regulation and significance (Fig. 5c). Of note, all GO terms were enriched with upregulated genes in infected *Gpr15*^*gfp/gfp*^ mice (red *z*-scores). Besides general terms—for example, ‘defense response’ and ‘immune system process’—we also found more specific terms, such as ‘cell migration’ and ‘cellular response to interferon-alpha’. Again, it became obvious that the difference between both genotypes was more pronounced on day 7. These results indicate that the cardiac inflammation as response to virus infection seems to be prolonged in the GPR15-deficient mice.

**Extended Data Fig. 4:**
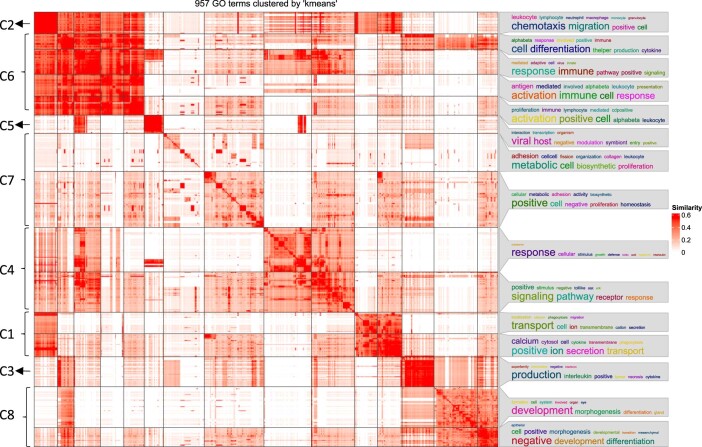
Similarity matrix of significant GO terms of the domain biological process. 957 GO terms that were significantly different on day 6 and, or on day 7 between both infected genotypes were clustered based on semantic similarity using the R package *simplify enrichment*. “Lin” method was used to calculate the similarity matrix for these GO terms. 15 semantic similarity clusters were determined (right) and were further summarized into 8 main clusters due to strong similarities (left, C1-C8). Source data

Next, we aimed to investigate the changes from day 6 to day 7 in more detail. Therefore, we selected all GO terms from the domain biological process (BP) that were significantly enriched with DEGs between both infected genotypes on day 6 (358 GO terms), on day 7 (782 GO terms) or on both days (183 GO terms). These GO terms were clustered by semantic similarity, as shown in Extended Data Fig. 4. The GO terms of each cluster were sorted based on their *P* value as follows: (1) not significant (NS), (2) significant (*P* < 0.05, gray) and (3) highly significant (*P* < 0.01, dark gray). As shown in Fig. 5d, the number of significantly and highly significantly enriched GO terms noticeably increased from day 6 to day 7. This can be observed in almost all clusters but is particularly clear for cluster 2 (‘chemotaxis/migration immune cells’) and cluster 6 (‘immune response/alphabeta T cells’). These changes of significance are displayed as alluvial plots in Fig. 5e. In cluster 2 (‘chemotaxis/migration immune cells’), only eight of 48 GO terms were significantly enriched on day 6. In contrast, 47 GO terms were significantly or highly significantly enriched with DEGs on day 7. Similarly, cluster 6 (‘immune response/alphabeta T cells’), containing 179 GO terms, revealed 62 significantly enriched GO terms on day 6, which was increased to 158 significantly enriched GO terms on day 7. In summary, significantly enriched GO terms regarding chemotaxis and T cell immune response became evident for day 7.

Due to these results, we examined the 7-day timepoint in more detail to identify important GO terms out of all three domains (BP, cellular component (CC) and molecular function (MF)). To identify the most prominently enriched GO terms, the 939 significantly enriched GO terms from day 7 were restricted by gene ratio > 0.1 and *q* < 0.001 and narrowed down using REVIGO to finally 25 GO terms, which were plotted in Fig. 5f. These 25 GO terms are almost exclusively related to the immune response to a viral infection and include genes significantly upregulated in infected *Gpr15*^*gfp/gfp*^ mice. Of note, many GO terms are included in clusters 2 and 6 (highlighted in bold). Particularly noteworthy are GO terms linked to chemotaxis and to T cells. So far, the GO analyses of RNA sequencing data showed marked upregulation of genes in GPR15-deficient mice on day 7 assigned to GO terms associated with chemotaxis of immune cells and T-cell-mediated immune response.

### GPR15 deficiency abolished chemotaxis of T cells

So far, the previous results point toward alterations in chemotaxis and cardiac inflammation, particularly associated with T cells, in GPR15-deficient mice during myocarditis. Based on those findings, we investigated the interactions of GPR15 and its two known ligands, GPR15L and the EGF-like domain 5 of thrombomodulin (TME5), in vitro.

**Fig. 6:**
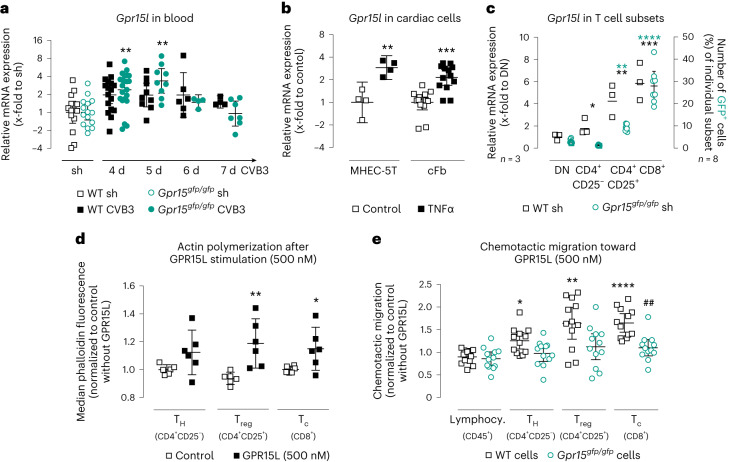
GPR15-expressing splenocytes and their interactions with the ligand GPR15L. **a**, *Gpr15l* gene expression in blood (*n* numbers stated in Fig. 2a). Ct values were normalized to *18S* and the corresponding sham controls (ΔΔCt). Significance was tested using an unpaired two-tailed *t*-test with Bonferroni correction. **b**, *Gpr15l* gene expression in MHEC-5T (25 ng ml^−1^, 5 h, *n* = 4 biological replicates) and cFBs (10 ng ml^−1^, 6 h, *n* = 13 biological replicates in three independent experiments) after TNFα stimulation. Ct values were normalized to *18S* and the corresponding untreated controls (ΔΔCt). Unpaired two-tailed *t*-test. **c**, Left *y* axis: splenocytes, pooled from 3–5 WT mice, were sorted into four CD45^+^ groups—CD4^+^CD25^−^ T_H_, CD4^+^CD25^+^ T_reg_, CD8^+^ T_C_ and DN cells—using FACS. *Gpr15* gene expression was determined in three independent experiments. Ct values were normalized to the mean of *Cdkn1b* and *18S* and to the DN cells (ΔΔCt). Repeated-measures one-way ANOVA. Right *y* axis: splenocytes from *Gpr15*^*gfp/gfp*^ mice (*n* = 8) were analyzed by flow cytometry. Percentage of GFP^+^ cells was determined in the same four groups (mean ± 95% CI). Repeated-measures one-way ANOVA. **d**, Evaluation of actin polymerization mediated by GPR15L. Isolated T cells (pooled from 2–3 WT mice) were incubated with 500 nM GPR15L for 180 s, immediately fixed and subsequently labeled and analyzed by flow cytometry. Actin polymerization was determined in two independent experiments. Median fluorescence intensity of six biological replicates was normalized to non-stimulated cells (mean ± 95% CI). Unpaired two-tailed *t*-test. **e**, GPR15L-mediated migration of primary splenocytes by Boyden chamber assays (*n* = 12) in three independent experiments. After 3 h, migrated cells were analyzed by flow cytometry. Results were normalized to cells passively migrated to media without GPR15L (mean ± 95% CI). Unpaired two-tailed *t*-test with Bonferroni correction. Gene expression data were plotted as 2^−ΔΔCt^ (geo-mean ± 95% CI). Significant, compared * to control or control of the same genotype, # between similarly treated groups of different genotypes. (*, **, ***, ****; *P* < 0.05, 0.01, 0.001, 0.0001). d, days; sh, sham. Source data

**Extended Data Fig. 5:**
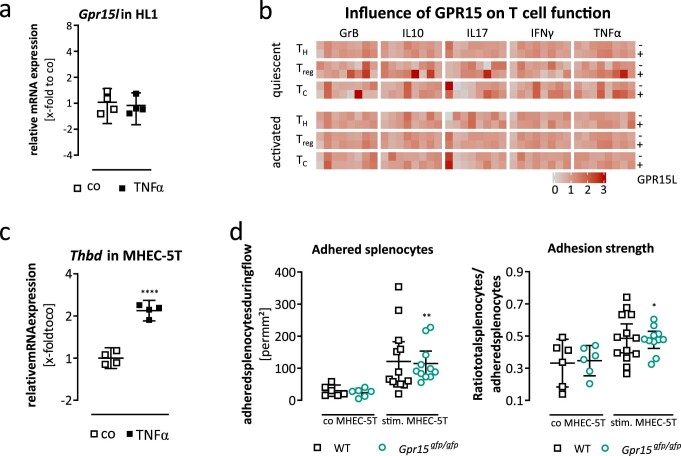
Adhesion assay and analysis of GPR15-mediated T cell functionality. (a) *Gpr15l* gene expression in HL1 cardiomyocytes after TNFα stimulation (10 ng/ml, 6 h, n = 4 biological replicates). Ct-values were normalised to *Cdnk1b* and the corresponding untreated control (ΔΔCt). Unpaired two-tailed t-test. (b) Quiescent or activated T cells were incubated in the presence or absence of the receptor agonist GPR15L (500 nM). Percentage of positive cells for the intracellular markers (GranzymeB (GrB), IL10, IL17, IFNγ, TNF-α) was quantified for three different T cell populations: CD4^+^CD25^-^: T helper cells (T_H_); CD4^+^CD25^+^: regulatory T cells (T_reg_); CD8^+^: cytotoxic T cells (T_C_). (c) Thrombomodulin (Thbd) expression after TNFα stimulation in MHEC‐5 T (25 ng/mL, 5 h, n = 4 biological replicates). Ct‐values were normalised to 18 S and the untreated control (ΔΔCt). Gene expression data were plotted as 2^-ΔΔCt^ (geo-mean ± 95% CI). Unpaired two‐tailed t‐test. (d) MHEC‐5 T monolayer were either cultured with growth medium alone or supplemented with 25 ng/mL TNFα for 24 h. Adhesion of primary splenocytes to MHEC‐5 T monolayer (n = 6-13 biological replicates in 3 independent experiments) was investigated during constant flow for 20 min. The adhesion strength of primary splenocytes was analysed after 5 min of flow pause to allow cells to adhere before flow was turned on to remove nonadherent splenocytes. Adherent splenocytes were counted via FIJI (mean ± 95% CI). Unpaired two‐tailed t‐test. Significant, compared * to control or control of the same genotype (*, **,****; *p* < 0.05, 0.01, 0.0001). Source data

Regarding the results of the GO analysis, which particularly highlighted chemotaxis, we hypothesized that GPR15 may function as a chemokine receptor mediating T cell homing during myocarditis. In addition to the receptor expression, we, therefore, reconsidered the blood samples of infected mice to evaluate the gene expression of the ligand *Gpr15l* in the acute phase of myocarditis. The *Gpr15l* expression was significantly increased in blood of GPR15-deficient mice at 4 days p.i. as well as 5 days p.i. (Fig. 6a). Next, we performed in vitro stimulation experiments on resident cardiac cells using the pro-inflammatory cytokine TNFα to investigate the regulation of *Gpr15l*. As shown in Fig. 6b, endothelial heart cells (MHEC-5T) and primary cardiac fibroblasts (cFBs), but not HL1 cells (Extended Data Fig. 5a), revealed an increased gene expression of *Gpr15l* after TNFα stimulation.

Subsequently, we investigated the impact of GPR15 on T cell chemotaxis. In a first approach, we treated WT T cells with the ligand GPR15L and quantified induced actin polymerization by phalloidin staining (Fig. 6d). Subsequent flow cytometry analysis revealed that T_C_ and T_reg_ cells significantly increased their actin polymerization in response to GPR15L treatment. Because actin polymerization is a key mechanism in the process of cell migration, we next tested the migration of lymphocytes in a Boyden chamber assay. Therefore, the chemotactic migration of WT or GPR15-deficient splenocytes toward the ligand GPR15L was examined. As depicted in Fig. 6e, the ligand GPR15L significantly increased the number of migrated T cells, with T_reg_ and T_C_ cells showing the highest effect. This chemotaxis was completely abolished in cells lacking the receptor GPR15.

Of note, both chemotaxis assays presented here indicate a T-cell-specific effect. Thus, we quantified the gene expression of *Gpr15* in different T cell subsets (Fig. 6c, left *y* axis). Although the T_H_ cells revealed only slightly higher *Gpr15* gene expression compared to double-negative (DN) cells, the expression in both T_reg_ and T_C_ cells was remarkably higher. Additionally, we used *Gpr15*^*gfp/gfp*^ splenocytes to quantify the number of GFP^+^ cells, here used as equivalent of GPR15 protein expression, within the different T cell subsets by flow cytometry (Fig. 6c, right *y* axis). In contrast to T_H_ cell populations, T_C_ and T_reg_ cell populations revealed significant increased number of GFP^+^ cells compared to DN lymphocytes, with T_C_ showing the highest number. This is in line with the *Gpr15* gene expression data.

To address whether GPR15 alters the functionality of T cells, isolated T cells were activated in the absence or presence of the receptor agonist GPR15L. As shown in Extended Data Fig. 5a, GPR15L itself did not significantly change the protein expression of various T cell activation markers. TME5, a domain of the transmembrane protein thrombomodulin on endothelial cells, is described as another GPR15 ligand^19^. Pro-inflammatory stimulation of endothelial heart cells using TNFα led to an increased gene expression of thrombomodulin (Extended Data Fig. 5b). To investigate the influence of GPR15 on leukocyte adhesion on endothelial cells, a flow assay on an endothelial monolayer was performed. As depicted in Extended Data Fig. 5c, the number of adherent splenocytes increased on TNFα-activated endothelial cells but was not different between WT and GPR15-deficient splenocytes. In terms of adhesion strength, on average around 50% of the splenocytes remained firmly attached to the stimulated endothelial cells, regardless of the presence of GPR15 (Extended Data Fig. 5b). These results indicate that GPR15 has no relevant impact on cell adhesion to endothelial cells.

Taken together, GPR15 mediated the chemotactic migration of T_reg_ and T_C_ cells toward its chemoattractant ligand GPR15L rather than contributing to leukocyte attachment before transendothelial migration or influencing T cell function. Therefore, our experiments strengthen the hypothesis that GPR15 is essential for the recruitment of T cells to the site of inflammation in the acute phase of CVB3-induced myocarditis.

### IFNγ reduced CVB3 replication in cardiomyocytes

**Fig. 7:**
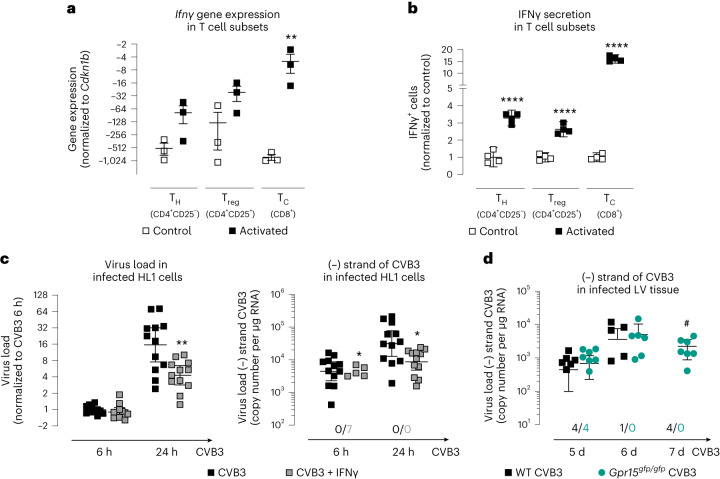
IFNγ in different T cell subtypes and its influence on virus load and replication in HL1 cells. **a**, Isolated T cells (pooled from four mice) were activated and sorted by FACS. *Ifn*γ gene expression was determined in three independent experiments. Ct values were normalized to *Cdkn1b*. Unpaired two-tailed *t*-test. Gene expression data were plotted as 2^−ΔCt^ (mean ± s.e.m.). **b**, Isolated T cells (*n* = 4 biological replicates, each pooled from 2–3 mice) were activated to determine IFNγ secretion. The number of IFNγ^+^ cells was normalized to the number of IFNγ^+^ cells in the respective control group (mean ± 95% CI). Unpaired two-tailed *t*-test. **c**, HL1 cells were treated with 100 ng ml^−1^ IFNγ for 20 h before infection with 0.5 MOI CVB3 for 1 h. IFNγ treatment was maintained during and after infection (*n* = 12 biological replicates in three independent experiments). HL1 cells were lysed 6 h or 24 h after CVB3 infection. To determine CVB3 virus load, isolated RNA was reversely transcribed by random primer. Ct values were normalized to *Hprt* and untreated CVB3-infected cells (6 h p.i.) (ΔΔCt). Unpaired two-tailed *t*-test with Bonferroni correction. 2^−ΔΔCt^ values were plotted (geo-mean ± 95% CI). To quantify virus replication, isolated RNA was reversely transcribed using the tagged (−) strand-specific primer. Copy number of the CVB3 minus strand was calculated based on a purified and quantified PCR product. The number of samples without detectable replication is shown below the dot plot. After 6 h, virus replication was not detectable in seven samples of the IFNγ-treated group (geo-mean ± 95% CI). Unpaired two-tailed *t*-test with Bonferroni correction. **d**, Virus replication was determined in LV tissue after 5 days, 6 days and 7 days p.i. (*n* numbers are shown in Fig. 2a). The number of samples without detectable replication is shown below the dot plot. Unpaired two-tailed *t*-test with Bonferroni correction (geo-mean ± 95% CI). Unpaired two-tailed *t*-test with Bonferroni correction. Significant, compared * to control, # between similarly treated groups of different genotypes. (*, **, ****; *P* < 0.05, 0.01, 0.0001). d, days; h, hours. Source data

Furthermore, we aimed to investigate the causes and subsequent effects of the lower IFNγ levels in the LV tissue of infected GPR15-deficient mice at day 5 p.i. To this end, we analyzed *Ifnγ* gene expression in and IFNγ secretion from activated T cell subtypes in vitro (Fig. 7a,b). Although the increase of *Ifnγ* gene expression after stimulation was significant only in T_c_ cells, IFNγ secretion was significantly increased in all T cell subtypes, with the increase being highest in T_C_ cells. This indicates that activated T_C_ cells are one of the main sources of IFNγ in the infected myocardium.

Next, we analyzed the influence of IFNγ on virus load and replication in infected HL1 cells (Fig. 7c). Therefore, IFNγ-treated HL1 cells were infected with 0.5 multiplicity of infection (MOI) CVB3. Although IFNγ-treated cells revealed no difference in virus load but less virus replication at 6 h p.i., at 24 h after infection both virus load and replication were decreased compared to untreated CVB3-infected cells.

Considering that *Ifnγ* gene expression was lower in GPR15-deficient mice 5 days p.i. (Fig. 2g), we returned to the animal experiment and examined virus replication in LV tissue after 5 days, 6 days and 7 days p.i. As shown in Fig. 7d, comparing infected WT and GPR15-deficient mice, virus replication in LV tissue was not different at 5 days and 6 days p.i. In contrast, virus replication was not detected at 7 days p.i. in any of the infected WT mice but was detected in all infected GPR15-deficient mice. This suggests a late consequence on virus replication of the lower *Ifnγ* expression in these mice at 5 days p.i.

## Discussion

In this study, we examined the consequences of GPR15 deficiency on development, progression and recovery in experimental CVB3-induced viral myocarditis. Our main findings are as follows: (1) GPR15 deficiency led to insufficient virus elimination accompanied by further aggravated disease severity and impaired cardiac function in the subacute phase of myocarditis. (2) Although similar on day 5 and day 6, GPR15-deficient mice exhibited higher cardiac virus load at day 7 p.i. (3) GPR15-deficient mice exhibited a delayed recruitment of T_reg_ cells and lower *Ifny* expression at day 5 p.i. but more pronounced inflammatory response at day 7 p.i. compared to WT mice. (4) Bulk RNA sequencing revealed that the response to virus did not decline from day 6 to day 7 in GPR15-deficient mice as observed in WT mice. Significant enrichment of upregulated DEGs in GO terms related to chemotaxis and T_C_ cells in GPR15-deficient mice on day 7 was shown. (5) GPR15 was highly expressed on T_reg_ and T_C_ cells, and its deficiency abolished chemotaxis of T cells toward GPR15L in vitro.

GPR15 was discovered owing to structural homology to known chemokine receptors^11^. In humans, *GPR15* is highest expressed in the intestine and lymphoid tissues and is found on T and B cells in various organs (Human Protein Atlas: GPR15; https://www.proteinatlas.org/ENSG00000154165-GPR15). In the murine intestine, GPR15 is preferentially expressed on distinct T cell subsets, particularly T_reg_ and T_C_ cells, whereas a very low number of T_H_ cells express GPR15 (ref. ^15^).

GPR15 appears to be a counter-regulator of inflammation under patho-physiological conditions;^26^ its knockout exacerbates skin^16^ and colon^15^ inflammation in mice. During colitis, the receptor is described as a T cell homing receptor especially for T_reg_ cells, but, in other organs, such as skin, it also regulates the migration of other T cell subsets during acute or chronic inflammatory states^18^. Furthermore, clinical cohort studies identified GPR15 as a major risk for cardiovascular diseases^27–29^. In various murine heart failure models, *Gpr15* expression was highly increased during the inflammatory stage of myocardial infarction and even more during acute viral myocarditis. Therefore, GPR15-deficient mice were investigated in the context of CVB3-induced viral myocarditis.

The non-susceptible C57BL/6J mouse strain used in this study develops acute myocarditis to eliminate the virus, resolves cardiac inflammation thereafter and, finally, recovers from myocarditis^21,22,30^. Kim et al.^15^ unveiled GPR15 as a homing receptor for T_reg_ cells during colitis and showed that GPR15 is not required for controlling the infection but, rather, for dampening the immune response. In the present study, we first analyzed the CVB3-induced myocarditis in the subacute phase at 16 days p.i. Starting with a similar virus load, the virus elimination was significantly impaired in GPR15-deficient mice. Although we determined virus persistence in most GPR15-deficient mice, they managed to dispel cardiac inflammation. However, cardiac function was impaired, accompanied by enhanced cardiac fibrosis, in GPR15-deficient mice.

Next, we extensively studied the key moment (5 days, 6 days and 7 days p.i.) when body weight development diverged between WT and GPR15-deficient mice, which may emphasize the deteriorated disease progression and severity in GPR15-deficent mice. WT and GPR15-deficient mice revealed equal virus loads in LV tissue at days 5 and 6 p.i., although, at day 5 p.i., the gene expression of *Ifnγ*, *Cd3* and *Foxp3* was significantly lower in GPR15-deficient mice. Lower *Foxp3* expression suggests delayed recruitment of T_reg_ cells. However, this conclusion is based only on gene expression data, as the barely abundant cell type of T_reg_ cells is hardly detectable by histology and flow cytometry. At day 6 p.i., both genotypes showed similar inflammatory responses in the cardiac tissue. In line with the divergent weight development, virus load and inflammatory response started to decline in infected WT mice at day 7 but remained consistently high in GPR15-deficient mice. We, therefore, investigated this turning point between day 6 and day 7 by RNA sequencing. GO term analyses uncovered several chemotaxis-related terms to be significantly enriched with highly upregulated genes in GPR15-deficent mice at 7 days p.i. Because GPR15 is described as a chemokine receptor^11,12^, especially on T cells, we assume that GPR15 deficiency causes a primarily delayed immune cell recruitment and, afterwards, a prolonged inflammatory response, resulting in an impaired outcome. Similarly, deficiency of other chemokine receptors, CCR5 or CX_3_CR1, leads to aggravated pathogen-induced myocarditis described by increased mortality or impaired cardiac function^31,32^.

Two ligands are known for GPR15: GPR15L, a soluble chemoattractant protein, and TME5, a subunit of the integral membrane protein thrombomodulin^18,19^. In our study, *Gpr15l* expression was upregulated in cardiac residential cells after pro-inflammatory stimulation facilitating T cell recruitment to the site of injury, as shown in other models^20^. In vitro, GPR15 deficiency abolished chemotactic migration of T_reg_ and T_C_ cells toward GPR15L, supporting the hypothesis that GPR15 acts as a chemokine receptor. This finding was further strengthened by increased actin polymerization after GPR15L treatment in T_reg_ and T_C_ cells. In addition, we investigated the influence of GPR15 on the adhesion of splenocytes to endothelial cells via the ligand TME5 expressed on the cell surface of endothelial cells and on cytokine production of T cells, whereby no influence of GPR15 could be proven.

Previous studies showed that both T_C_ and T_reg_ cells have an impact on the outcome of CVB3-induced myocarditis. Adoptive T_reg_ transfer during the inflammatory phase of viral myocarditis or before induction of myocarditis protects the heart against inflammatory damage^33,34^. Here, delayed recruitment of T_reg_ cells is followed by prolonged inflammation and more severe cardiac damage. Henke et al.^35^ showed that depletion of T_C_ cells led to improved survival in the early phase of CVB3-induced myocarditis but increased virus load, emphasizing their important role in virus elimination^35^. Here, we show that activated T_C_ cells highly increase their IFNγ secretion and that IFNγ reduces virus load and virus replication in CVB3-infected cardiomyocytes in vitro. Therefore, we assume that T_C_ cells are one of the main sources of the increased *Ifnγ* expression in vivo and that the lower *Ifnγ* expression in GPR15-deficient mice might cause the impaired virus elimination. However, the exact cause of the decreased *Ifnγ* expression 5 days p.i. in the LV tissue of GPR15-deficient mice cannot be elucidated here. It can be assumed that the recruitment not only of T_reg_ cells but also that of T_C_ cells is impaired by GPR15 deficiency. This would correspond to our in vitro results, according to which T_C_ cells react strongly chemotactically to the ligand GPR15L. Furthermore, IFNγ has been shown not only to be an anti-viral agent in itself^36^ but also to reduce fibrosis and to prevent the development of severe chronic myocarditis^37^. The here-observed late sequela of myocarditis by GPR15 deficiency might be traced back to delayed recruitment of T_reg_ cells, but possibly also T_C_ cells, into the virus-infected myocardium.

In conclusion, we identified GPR15 as a chemokine receptor crucial for a better outcome after acute viral myocarditis. In vitro, GPR15 facilitates GPR15L-mediated recruitment of T cells, particularly T_C_ cells essential for virus elimination and T_reg_ cells essential for dampening cardiac inflammation. In myocarditis, GPR15 deficiency leads to delayed migration of T_reg_ cells to the infected myocardium, resulting in inflammatory-induced cardiac injury from day 7 p.i. In addition, expression of *Ifnγ* was delayed in LV tissue of GPR15-deficient mice, which might lead to ongoing virus replication in the early phase of myocarditis and, thus, virus persistence later. In line with Kim et al.^15^, GPR15 is a homing receptor for T_reg_ cells and important for dampening the cardiac inflammation. Moreover, the GPR15-mediated migration of T_C_ cells is essential for virus elimination in viral myocarditis to prevent progression to heart failure.

## Methods

### Animal model

For the present study, we employed the previously described knock-in mouse strain (B6; 129P2-*Gpr15*^*tm1.1Litt*^/J) in which the endogenous *Gpr15* gene was replaced by the sequence of the GFP^15^. Thus, these mice can be used as *Gpr15* knockouts. Mice were bred on B6 background, and male littermates or offsprings from littermates identified as homozygous B6 WT or GPR15-deficient mice were used for subsequent experiments.

Additionally, cDNA from LV tissue obtained by previous studies was analyzed^38^. Three murine heart failure models were investigated: (1) chronic hypertension induced by continuous infusion of angiotensin II, (2) myocardial infarction induced by permanent occlusion of the left anterior descending artery and (3) CVB3-induced myocarditis^21,39,40^.

#### Experimental viral myocarditis

As depicted in Figs. 1b and 2a, male B6 WT (*Gpr15*^+/+^) or GPR15-deficient (*Gpr15*^gfp/gfp^) mice were used at the age of 7–9 weeks to induce experimental viral myocarditis^21^. Therefore, 5 × 10^5^ plaque-forming units (PFU) of CVB3 (Nancy strain) were injected intraperitoneally under short-time CO_2_/O_2_ anesthesia. Sham controls were treated equally, but saline was used for injection. On day 4, blood was drawn via facial vein to prove infection. The body weight was monitored daily. If blood sampling did not yield sufficient RNA to prove viremia, but virus infection was proven in the LV tissue later, mice were included into the analysis. CVB3-infected mice that did not show virus load in blood at day 4 p.i. or in LV tissue later were excluded from the study. Timelines studying acute or subacute phase of myocarditis are depicted in Figs. 2a and 1b, respectively. The *n* numbers of included CVB3^+^ mice and the original *n* numbers are shown separately for each experiment and were calculated using G*Power (version 3.1.9.7).

To study the acute phase, mice were killed 5 days, 6 days or 7 days p.i. Blood was taken from the beating heart and snap frozen in liquid nitrogen. After heart explantation, atria were removed, and hearts were transversally cut to create a cross-section that was then fixed in 10% neutral-buffered formalin solution for 24 h. The other part of the LV tissue was immediately snap frozen in liquid nitrogen and stored at −80 °C. In addition, lymph nodes were harvested for the 5-day timepoint and snap frozen in liquid nitrogen. To study the subacute phase, 16 days p.i., cardiac function was recorded by hemodynamic measurements. Heart and blood were collected and processed as described above.

All mice were housed under pathogen-free conditions in the animal facility of the University Medical Centre Hamburg-Eppendorf at 22 °C with ad libitum access to water and standard laboratory chow diet. All animal experiments were approved by the local bioethics committee of Hamburg, Germany (G13/115, G15/060, N060/2020, ORG821 and ORG1068) and conform to the *Guide for the Care and Use of Laboratory Animals*, published by the US National Research Council (8th edition, revised 2011)^41^.

#### Hemodynamics

A PV loop system (ADV500, Transonic) was used for hemodynamic measurements in closed-chest approach^39,42^. Mice were anesthetized using urethane (0.8–1.2 g kg^−1^ body weight) accompanied by buprenorphine analgesia (0.1 mg kg^−1^ body weight). First, a tracheotomy was performed for artificial ventilation. Subsequently, a pressure–conductance catheter (1.2 F, Transonic) was inserted in the right carotid artery and carefully pushed forward into the LV. The catheter’s position inside the ventricle was optimized until rectangular-shaped loops were obtained. PV loops were recorded under short-time apnea. The inferior caval vein (ICV) was occluded by gentle compression during the PV loop measurements. Using the wash-in technique, a bolus of hypertonic saline (10%) was injected into the left jugular vein to estimate the volume^43^. Data were acquired using iox2 (version 2.9.5.73, Emka Technologies). Subsequent analyses of PV loops were performed in LabChart 7.3 Pro (ADInstruments). For baseline analysis, 5–10 consecutive loops during end-expiratory ventilation pause were selected to calculate preload-dependent parameters. Preload-independent parameters were analyzed by selecting loops during ICV occlusion^43^. Mice with too deep anesthesia or excessive bleeding during the measurement were excluded from the analysis.

### RNA-based analyses

#### RNA isolation

Total RNA was isolated from snap-frozen tissue samples using QIAzol lysis reagent and further purified using an miRNeasy Mini Kit (Qiagen) according to the manufacturer’s protocol. Previously, frozen tissue was disrupted by stainless steel beads in a 2 -ml tube filled with QIAzol using a tissue lyser II (Qiagen). To obtain total RNA from blood and cells, an RNeasy Mini Kit (Qiagen) was used according to the manufacturer’s protocol. To avoid genomic DNA contamination within the isolated RNA, DNase-I (Qiagen) was applied directly on the column during the purification protocol. RNA concentration was determined using a NanoDrop 2000c spectrophotometer (Thermo Fisher Scientific). RNA was stored at −80 °C.

#### Reverse transcription and gene expression analysis

RNA was reversely transcribed into cDNA using a high-capacity cDNA kit (Life Technologies). Depending on the underlying experiment, we used 1 µg of RNA from tissue samples, 0.1 µg from blood and 0.25 µg from cell culture experiments for cDNA synthesis. Reverse transcription was carried out at 37 °C for 2 h, followed by an inactivation step of 5 min at 85 °C. The resulting cDNA was diluted to a final working concentration of 10 ng µl^−1^ for tissue samples or 1.25 ng µl^−1^ for blood and cell culture samples.

Quantitative real-time PCR was performed to assess gene expression of target genes using 2.5 µl of gene expression master mix (Thermo Fisher Scientific) and 0.25 µl of gene expression assay (Supplementary Table 1). CVB3 load was determined using primers and probe (Supplementary Table 2)^24,44^. A volume of 1 µl of cDNA was used as template in a final volume of 5 µl. Each sample was analyzed in duplicates. Real-time PCR was performed on a QuantStudio 7 Flex or QuantStudio 7 Pro system (Thermo Fisher Scientific) using QuantStudio software version 1.3 or Design and Analysis software 2.6.0, respectively. Gene expression of *Cdkn1b* and/or *18S* or *Hprt* was determined as endogenous controls, and ΔCt values were calculated for the target genes. The formula 2^−ΔCt^ was used to calculate absolute gene expression, and obtained values were plotted as x-fold to *Cdkn1b* or *18S* or the mean of both or *Hprt*. For normalization, mean of ΔCt values of the respective control group was used to calculate ΔΔCt values. Relative gene expression data were determined using the formula 2^−ΔΔCt^ and plotted as x-fold to the respective control^45^.

#### Detection of virus replication

Virus replication was determined by strand-specific reverse transcription of the intermediate minus RNA strand of CVB3, which is essential for production of the viral genome of viral progeny. Therefore, isolated total RNA was reversely transcribed into strand-specific cDNA using a high-capacity cDNA kit (Life Technologies) in the presence of 1 µM minus strand-specific primer (RT-CVB3_SE_tagged; 5′-TGAGATAATTGCCCTGAATGCGGCTAATCC-3′, TIB Molbiol). To avoid subsequent detection of non-specific cDNA synthesis, the minus strand-specific primer for reverse transcription was tagged by adding 11 nucleotides at the 5′ end (Supplementary Table 3). In addition, cDNA synthesis was performed in the absence of the minus strand-specific primer as an additive control of specificity. Subsequently, the tagged minus strand-specific cDNA was quantified with specific primers amplifying only tagged cDNA by real-time PCR as described above. Copy number was calculated using a purified and quantified PCR product, because endogenous control genes were not available.

#### MACE RNA sequencing analysis

Massive analysis of cDNA ends (MACE) is a 3′ mRNA sequencing method based on the analysis of Illumina reads derived from fragments that originate from 3′ mRNA ends^46^. Thirty-one RNA samples from the acute myocarditis model were used for RNA sequencing (WT sham (*n* = 6) / *Gpr15*^*gfp/gfp*^ sham (*n* = 6) / WT CVB3 (6 days *n* = 6; 7 days *n* = 3) / *Gpr15*^*gfp/gfp*^ CVB3 (6 days *n* = 5; 7 days *n* = 5)). RNA samples were processed by GenXPro using the MACE Kit (version 2) according to the manufacturerʼs manual. In brief, RNA was fragmented, and polyadenylated mRNA was enriched and amplified by competitive PCR after poly(A)-specific reverse transcription and template-switch-based second-strand syntheses. Duplicate reads as determined by the implemented unique molecular identifiers (TrueQuant IDs) were removed from the raw dataset. Low-quality sequence bases were removed by Cutadapt software (version 2.3)^47^, and poly(A) tails were clipped by an in-house Python script. The reads were mapped to the mouse genome (mm10), and transcripts were quantified by HTSeq. Differential gene expression was determined using DESeq2 (version 1.20)^48^ and plotted as volcano plots using Graph Pad Prism (GraphPad Software). For selected DEGs, gene expression of all replicates was visualized as a heat map. A pseudo-count was introduced for every not-expressed gene to allow logarithmic calculation after normalization. The normalized gene count was standardized to the mean of the normalized gene count of the respective sham group and plotted as log_2_ FC. For data visualization, the R statistical software (version 3.6.3, R Foundation for Statistical Computing) tool ComplexHeatmap was used^49^.

#### GO enrichment analysis

GO enrichment analysis was performed using the R package topGO (version 2.42.0)^50^. GO annotation data were based on ENSEMBL. Enrichment of GO terms was calculated by Fisher’s exact test based on DEGs (*P* < 0.05 and FC of at least ±1.5). The results were visualized using different R packages. Clustering of GO terms was performed with the R package simplifyEnrichment (version 1.0.0)^51^. First, the semantic similarity measurement was calculated based on the method of Lin^52^. The resulting similarity matrix was then clustered by the *k*-means method. Based on their *P* values, GO terms of particular clusters were split into three categories and plotted as alluvial plots created with ggforce (version 0.3.3)^53^ and ggplot2 (version 3.3.3)^54^. The latter was also used to generate dot plots. Color of the symbol is based on the GO term’s *z*-score. It is calculated for each GO term by taking the number of upregulated DEGs, subtracting the number of downregulated DEGs and dividing this number by the square root of the number of annotated genes. Whether most DEGs in this GO term are upregulated or downregulated in *Gpr15*^*gfp/gfp*^ mice is indicated by the color gradient from red to blue, respectively. The chord plot was generated using GOChord from the R package GOplot (version 1.0.2)^55^. It represents the association of genes to GO terms. The chord plot also visualizes the direction of regulation for depicted genes, with red representing upregulation and blue representing downregulation in *Gpr15*^*gfp/gfp*^ mice.

### Histological analyses

Cross-sections of murine hearts were fixed in 10% neutral-buffered formalin solution, dehydrated and embedded in paraffin (formalin-fixed paraffin-embedded (FFPE)) afterwards. Then, 4-µm-thick cross-sections were cut using a microtome. For all staining methods, FFPE sections were deparaffinized and rehydrated in a descending ethanol series.

#### Picrosirius red staining

Picrosirius red (PSR) staining was used to assess fibrosis of the cardiac tissue. Rehydrated slides were incubated for 90 s in Mayer’s hemalum solution (Merck Millipore) diluted 1:1 with distilled water to stain acidic structures and then blued in tap water at approximately 60 °C for 10 min. The basic tissue structures were counterstained by incubation for 60 s in a 1% eosin solution (Merck Millipore) slightly acidified with glacial acetic acid (Roth). For collagen staining, a PSR stain kit (Polysciences) was used according to the manufacturer’s protocol. Then, sections were dehydrated using an ascending ethanol series, followed by incubation in xylene substitute. Finally, the sections were covered with EUKITT (ORSAtec).

#### Chromogenic immunohistochemistry

After dewaxing and inactivation of endogenous peroxidases (PBS/3% hydrogen peroxide), antigen retrieval was performed using the Ventana BenchMark XT machine. Sections were incubated with anti-CD3 or anti-CD8 antibody (Supplementary Table 4) for 32 min. Anti-Rabbit Histofine Simple Stain MAX PO conjugated with universal immunoperoxidase polymer (medac-diagnostika) was used as secondary antibody. Detection of secondary antibody was performed with an ultraView Universal DAB Detection Kit (Ventana). For subsequent counterstaining, Hematoxylin Ventana Roche and Bluing Reagent Ventana Roche were used. Regarding chromogenic immunohistological staining and subsequent quantification of CD3^+^ and CD8^+^ T cells in LV tissue: for one GPR15-deficient CVB3-infected mouse (5 days), FFPE tissue was not available. Furthermore, for one GPR15-deficient CVB3-infected mouse (5 days) and one WT CVB3-infected mouse (5 days), CD8 staining was not successful.

#### RNAscope

An RNAscope Multiplex Fluorescent v2 Reagent Kit (323100, ACD) or an RNAscope 2.5 High Definition Reagent Kit-RED (322350, ACD) was used to perform in situ hybridization to detect CVB3 plus strand RNA on FFPE sections of ventricular tissue^56,57^. In brief, sections were incubated for 1 h at 60 °C and deparaffinized by xylene and 100% ethanol. To quench internal peroxidase activity, sections were incubated with hydrogen peroxide incubation for 10 min, followed by target retrieval at 95 °C for 10 min. Sections were permeabilized with Protease Plus at 40 °C for 30 min. Probes were incubated for 2 h at 40 °C, followed by RNAscope amplification steps. For subsequent immunofluorescent staining, sections were blocked with 3% BSA/TBS at room temperature for 2 h. Primary anti-troponin T antibody was incubated overnight at 4 °C in 1% BSA/TBS. After washing, sections were incubated with Alexa Fluor 488–coupled secondary antibody and Alexa Fluor 633–coupled wheat germ agglutinin (WGA) for 2 h at room temperature. The slides were mounted in DAPI Fluoromount-G. For chromogenic signal detection, nuclei were counterstained using hematoxylin. More detailed information about antibodies, probes and reagents is provided in Supplementary Table 5.

#### Microscopy

Images of PSR or chromogenic immunohistochemistry staining were captured using a BZ 9000 microscope (Keyence) with ×10 CFI PL APO Lbd. (NA = 0.45), ×20 CFI PL APO Lbd. (NA = 0.75) or ×60 CFI PL APO Lbd. H (NA = 1.4) objective and processed with BZ II Analyzer software. Fibrotic areas were quantified using FIJI (version 2.14.0). To quantify positive immune cells, tissue area of 2–3 cross-sections per mouse was measured with QuPath (version 0.4.3)^58^, whereas positive cells were counted manually. To capture confocal images, a Leica TCS SP5 confocal microscope (Leica Microsystems) with ×40 HCX PL APO CS (NA = 1.3) and ×63 HCX PL APO Lbd. Bl. oil (NA = 1.4–0.6) objectives was used. A maximum projection of three-dimensional images was created using Leica LAS AF software over the full range of the signal.

### Cell-based analyses

#### Murine cardiac fibroblasts

Primary murine cardiac fibroblasts were obtained by multiple repeated digestions of minced LV tissue from male WT B6 mice (10–12 weeks old) using 0.1 mg ml^−1^ Liberase (Roche)^59,60^. Obtained cardiac fibroblasts were resuspended in DMEM (Pan) containing 20% FCS (Thermo Fisher Scientific) and 100 U ml^−1^ penicillin–streptomycin (Thermo Fisher Scientific) and rapidly attached to cell culture flasks. Cells were kept in a humidified atmosphere at 37 °C, with 5% CO_2_ and 95% air.

#### Isolation of splenocytes and T cells

Primary lymphocytes from spleen were isolated from male WT and GPR15-deficient B6 mice at 8–16 weeks of age^39^. Spleen was removed, kept in ice-cold DPBS and mashed through a 70-µm cell strainer. Cells in the flow-through were collected, washed with DPBS and passed through a 40-µm cell strainer. For purification, cell suspension was carefully layered over 3 ml of Histopaque-1077 (Sigma-Aldrich) and centrifuged at 400*g* for 30 min. The splenocytes, contained in the interface, were aspirated, washed with DPBS and finally resuspended in RPMI VLE 1640 media (Pan) supplemented with 0.5% BSA. Before subsequent experiments, splenocytes were rested for 2 h in a humidified atmosphere at 37 °C, with 5% CO_2_ and 95% air. In case of subsequent T cell isolation by magnetic cell separation, Pan T Cell Biotin-Antibody Kit II (Milteny Biotec) was used according to the manufacturer’s protocol.

#### Chemotaxis assay

Freshly isolated primary splenocytes were used for a Boyden chamber assay. Cell culture inserts with 3 µm pore size (353492, Falcon) were coated with 50 µl of fibronectin (10 µg ml^−1^) at 37 °C for 1 h. Fibronectin solution was removed, and inserts were washed once with DPBS and dried at room temperature. Next, 1 × 10^6^ splenocytes in 250 µl of medium were added to the inserts and settled for 30 min. Wells of suited companion plate (Falcon) were filled with 700 µl of media as negative control or media supplemented with 500 nM GPR15L (25-78, Phoenix Pharmaceuticals). Inserts containing splenocytes were applied to the wells of the companion plate. Cell migration was allowed for 3 h under cell culture conditions. Thereafter, inserts were removed to access the lower chamber containing the migrated cells. To determine the initial population, splenocytes were added directly to 700 µl of medium without an interjacent insert and used to calculate the percentage of migration.

Migrated splenocytes were analyzed using flow cytometry analyses (Supplementary Fig. 1). First, splenocytes were blocked with 2% rat serum (STEMCELL Technologies) and 500 ng ml^−1^ anti-CD16/CD32 monoclonal antibody (clone 2.4G2, Bio X Cell) for 5 min at room temperature. Next, splenocytes were incubated with the staining master mix (Supplementary Table 6). Pacific Orange was used for live/dead discrimination. After washing once with DPBS, splenocytes were resuspended in 500 µl of DPBS for flow cytometry analysis (LSRFortessa, BD Biosciences, using FACSDiva software (version 9.0.1)). Single-stained controls were used for compensation. Flow cytometry data were analyzed using FCSalyzer software (version 0.9.22-alpha). The gating strategy for this analysis is shown in Supplementary Fig. 1.

#### Analysis of GPR15-expressing T cells

Using splenocytes isolated from WT mice, *Gpr15* expression of T cell populations was examined on transcriptomic level. WT splenocytes were sorted by fluorescence-activated cell sorting (FACS) (Aria IIIu, BD Biosciences, using FACSDiva software (version 9.0.1)) and stained with the master mix (Supplementary Table 6) as described in section 5.4.3. Cells were sorted into tubes filled with 1 ml of RLT buffer containing 1% β-mercaptoethanol for immediate cell lysis. RNA isolation and gene expression analyses were performed as described in section 5.2. GFP fluorescence as equivalent for GPR15 expression of T cell populations was examined using flow cytometry. Therefore, *Gpr15*^*gfp/gfp*^ splenocytes were stained as described above. GFP^+^ cells were counted during the flow cytometry analysis. The gating strategy is shown in Supplementary Fig. 2.

#### Actin polymerization assay

Isolated T cells were incubated with or without 500 nM GPR15L for 180 s and immediately fixed in 4% paraformaldehyde at room temperature for 20 min. Extracellular staining master mix (Supplementary Table 7) was added and incubated for 30 min at 4 °C in the dark. Cells were washed in DPBS supplemented with 10% FCS and subsequently fixed with IC Fixation Buffer (eBioscience) for 30 min at 4 °C. Afterwards, cells were washed in permeabilization buffer (eBioscience) and incubated in 1× Phalloidin-iFluor 488 (Abcam) diluted in permeabilization buffer for 30 min at 4 °C in the dark. Cells were washed again in permeabilization buffer, fixed with CellFIX (BD Biosciences) and acquired in a FACSCanto (BD Biosciences, using FACSDiva software (version 9.0.1)). Phalloidin median fluorescence intensity of different T cell populations was analyzed with FlowJo software (version 10.8.1). The respective gating strategy is shown in Supplementary Fig. 3.

#### IFNγ secretion assay

Isolated T cells were activated with Dynabeads Mouse T-Activator CD3/CD28 (Thermo Fisher Scientific). In brief, Dynabeads were incubated with T cells in a 1:1 ratio for 12 h in a 24-well plate under cell culture conditions. After removal of Dynabeads, IFNγ secretion was detected using a Mouse IFNγ Secretion Assay (Milteny Biotec) as described in the user manual. For subsequent flow cytometric analysis, cells were stained with staining master mix (Supplementary Table 8) and acquired in a FACSCanto II (BD Biosciences). IFNγ secretion was analyzed for different cell populations using FlowJo software (version 10.8.1). The respective gating strategy is shown in Supplementary Fig. 4.

#### *Ifnγ* gene expression in T cell subsets

Isolated T cells were activated with Dynabeads Mouse T-Activator CD3/CD28 as described in the previous section. After removal of Dynabeads, cells were stained with the staining master mix (Supplementary Table 8) and sorted by FACS (Aria III, BD Biosciences, using FACSDiva software (version 9.0.1)). Cells were sorted into tubes filled with 2–3 ml of RLT buffer containing 1% β-mercaptoethanol for immediate cell lysis. RNA isolation and subsequent TaqMan gene expression analyses were performed as described in section 5.2.

#### IFNγ treatment of CVB3-infected HL1 cells

HL1 cells (Merck, SCC065) were plated on 24-well plates and grown until confluence. Then, cells were stimulated with 100 ng ml^−1^ IFNγ (PeproTech) in RPMI VLE 1640 medium (0.5 % FCS and 100 U ml^−1^ penicillin–streptomycin) for 20 h in the incubator (37 °C, 5% CO_2_ and 95% air). Subsequently, cells were infected with 0.5 MOI CVB3. After 1 h, virus suspension was removed, and cells were further incubated for 23 h. Cells were lysed with 350 µl of RLT buffer containing 1% β-mercaptoethanol. RNA isolation and subsequent gene expression analyses were performed as described in section 5.2.

#### T cell activation assay

Isolated T cells were activated with Dynabeads Mouse T-Activator CD3/CD28 (Thermo Fisher Scientific) and/or 500 nM GPR15L (25-78, Phoenix Pharmaceuticals) as described in previous sections. For subsequent FACS analysis, beads were removed magnetically, and T cells were resupended in DPBS supplemented with 10% FCS. Next, extracellular staining master mix (Supplementary Table 9) was added and incubated at 4 °C for 30 min in the dark. Cells were washed in DPBS supplemented with 10% FCS and subsequently fixed with IC Fixation Buffer (eBioscience) at 4 °C for 30 min. Afterwards, cells were washed in permeabilization buffer (eBioscience), and the staining master mix for intracellular staining (Supplementary Table 10) was added and incubated at 4 °C for 45 min in the dark. Once again, cells were washed in permeabilization buffer, fixed with CellFIX (BD Biosciences) and acquired in an LSRFortessa (BD Biocencies). Positive cells for the intracellular markers (granzyme B, IL-17, IFNγ and TNFα) were counted for different T cell populations using FlowJo software (version 10.8.1). The respective gating strategy is shown in Supplementary Fig. 5.

#### Flow adhesion assay

In vitro flow adhesion assay was used to determine the attachment of splenocytes to endothelial cells. First, 100 µl of cell suspension containing 5 × 10^4^ MHEC-5T cells (DSMZ, ACC 336) was injected into the channel of μ-slides (0.4 Luer ibiTreat, ibidi)^61^. All subsequent steps were performed in a humidified atmosphere at 37 °C, with 5% CO_2_ and 95% air. After allowing cells to adhere for 4 h, ports were filled with growth medium (DMEM (Pan), 10% FCS, 100 U ml^−1^ penicillin and 100 µg ml^−1^ streptomycin), and slides were placed on a shaker with slow speed to ensure constant medium exchange. Approximately 12 h later, growth medium was carefully replaced by starvation medium (DMEM and 0.5% FCS) for 6–8 h. To enhance thrombomodulin expression, cells were stimulated with 25 ng ml^−1^ recombinant murine TNFα (PeproTech) in starvation medium for 24 h. Splenocytes were stained in medium supplemented with 2 µM CellTracker Red CMTPX (Thermo Fisher Scientific) for 30 min and rested for 2 h. Then, 1 × 10^7^ stained splenocytes were resuspended in 500 µl of medium and applied to the in vitro flow adhesion assay.

We used ibidi perfusion sets (WHITE: length 50 cm, ID 0.8 mm, 10-ml reservoirs) connected to a fluidic unit (ibidi, 10902), which was attached to a pump building up a continuous flow. First, the perfusion set was filled with pre-warmed 5 ml of RPMI VLE 1640 medium containing 0.5% BSA and attached to the μ-slide containing the endothelial monolayer, and air bubbles were removed. Next, the μ-slide was placed in an incubation unit (Tokai Hit, 5% CO_2_ and 37 °C) inside a fluorescence microscope (BZ-9000, Keyence). The fluidic unit was placed in an incubator (37 °C and 5% CO_2_). To start the flow adhesion assay, unidirectional flow with a shear stress of 1 dyne per cm^2^ was applied, and splenocytes were added to the perfusion set. To record the adhesion of splenocytes to the MHEC-5T monolayer, images were taken every 30 s for 20 min using bright-field and red fluorescence channel (tetramethylrhodamine (TRITC)). To investigate the adhesion strength, flow was switched off for 5 min. Subsequently, flow was switched on to remove not-adhered splenocytes, and images were captured. Adhered splenocytes were counted with FIJI software (version 2.14.0). The ratio between the total number of splenocytes and adherent splenocytes was calculated to analyze adhesion strength.

### Statistics

Gene expression data (2^−ΔCt^ and 2^−^^ΔΔCt^ values) are displayed with geometric mean ± 95% confidence interval (CI). All other data are presented as mean ± 95% CI, unless otherwise stated. All statistical analyses were performed using Gaussian normally distributed data. Hence, the statistics for the gene expression analyses were calculated with ΔCt or ΔΔCt values. In general, statistical comparison of two groups was performed using the unpaired two-tailed *t*-test with *P* < 0.05 considered as statistically significant. If more than two groups from both genotypes, WT and *Gpr15*^*gfp/gfp*^, were compared, Bonferroni correction was additionally used to adjust for multiple comparisons. If more than two groups from one genotype, either WT or *Gpr15*^*gfp/gfp*^, were compared, ordinary one-way ANOVA or repeated-measures one-way ANOVA (matched data) was used, each corrected with the Holm–Sidak method. To test for significance of daily weight measurements, multiple *t*-tests with Holm–Sidak correction were used. Fisher’s exact test was used for the analysis of contingency tables. Significance of GO term enrichment was calculated by Fisher’s exact test based on DEGs (*P* < 0.05 and FC > +1.5 or FC < −1.5). Adjusted *P* values (*q* values) of GO terms were determined using the Benjamini–Hochberg correction. All data were analyzed using GraphPad Prism 6 (versions 6.07 and 9.5.1) or R studio (version 4.0.2).

### Reporting summary

Further information on research design is available in the Nature Portfolio Reporting Summary linked to this article.

### Supplementary Information

Supplementary Information Supplementary Figs. 1–5 and Supplementary Tables 1–10

Reporting Summary

### Source data

Source Data Fig. 1–7 and Extended Data Fig. 1–5 Source data and exact *P* value for comparisons

## Data Availability

RNA sequencing data are available under GSE248521. Source data of all main figures are provided.

## Code availability

The code used for processing of RNA sequencing data (poly(A) tail clipping) is available at https://github.com/AGLindner/GPR15-RNA-Sequencing.
