# Author Correction: GPR15-mediated T cell recruitment during acute viral myocarditis facilitated virus elimination and improved outcome

**DOI:** 10.1038/s44161-024-00540-x

**Published:** 2024-08-23

**Authors:** Bastian Stoffers, Hanna Wolf, Lucas Bacmeister, Svenja Kupsch, Tamara Vico, Timoteo Marchini, Maria A. Brehm, Isabell Yan, P. Moritz Becher, Armin Ardeshirdavani, Felicitas Escher, Sangwon V. Kim, Karin Klingel, Paulus Kirchhof, Stefan Blankenberg, Tanja Zeller, Dennis Wolf, Ingo Hilgendorf, Dirk Westermann, Diana Lindner

**Affiliations:** 1 Department of Cardiology and Angiology, University Heart Center Freiburg-Bad Krozingen, Faculty of Medicine, University of Freiburg, Freiburg, Germany; 2 DZHK (German Centre for Cardiovascular Research), partner site Hamburg/Kiel/Lübeck, Hamburg, Germany; 3 Department Digital Health Sciences and Biomedicine, School of Life Sciences, University of Siegen, Siegen, Germany; 4 Department of Cardiology, University Heart & Vascular Centre Hamburg, University Medical Centre Hamburg-Eppendorf, Hamburg, Germany; 5 DZHK (German Centre for Cardiovascular Research), partner site Berlin, Berlin, Germany; 6 Institute for Cardiac Diagnostics and Therapy, Berlin, Germany; 7 Deutsches Herzzentrum der Charité, Department of Cardiology, Angiology and Intensive Care Medicine, Campus Virchow Klinikum, Berlin, Germany; 8 Department of Microbiology and Immunology, Sidney Kimmel Medical College, Thomas Jefferson University, Philadelphia, PA USA; 9 Cardiopathology, Institute of Pathology and Neuropathology, University Hospital Tübingen, Tübingen, Germany

**Keywords:** Cardiovascular diseases, Infectious diseases, Inflammation

Correction to: *Nature Cardiovascular Research* 10.1038/s44161-023-00401-z, published online 27 December 2023.

This article was originally published under standard Springer Nature license (© The Author(s), under exclusive licence to Springer Nature Limited). It is now available as an open-access paper under a Creative Commons Attribution 4.0 International license, © The Author(s). The error has been corrected in the HTML and PDF versions of the article.

