# Supplementary material for: GPR15-mediated T cell recruitment during acute viral myocarditis facilitated virus elimination and improved outcome
Source: Nat Cardiovasc Res. 2023 Dec 27;3(1):76–93. doi: 10.1038/s44161-023-00401-z (PMC11357984; doi:10.1038/s44161-023-00401-z)
Supplement: Supplementary file 1 — Supplementary Figs. 1–5 and Supplementary Tables 1–10 [file 44161_2023_401_MOESM1_ESM.pdf]

nature cardiovascular research

Supplementary information

https://doi.org/10.1038/s44161-023-00401-z

GPR15-mediated T cell recruitment during acute viral myocarditis facilitated virus elimination and improved outcome

In the format provided by the authors and unedited

Table of contents

1 EXPANDED MATERIAL AND METHODS 2

1.1 Gene expression analysis using TaqMan 2

1.2 Chromogenic Immunohistochemistry 5

1.3 RNAscope *in situ* hybrisation 6

1.4 Chemotaxis-assay and analysis of GPR15 expressing T cells 7

1.5 Actin polymerisation assay 10

1.6 IFNγ secretion assay 12

1.7 T cell activation assay 14

1 Expanded Material and Methods

1.1 Gene expression analysis using TaqMan

Supplementary Table 1: Gene expression assays purchased from Thermo Fisher Scientific.

Gene	Gene full name	Assay ID
18s	Eukaryotic 18S rRNA	Hs99999901_s1
Ccl2	Chemokine (C-C motif) ligand 2	Mm99999056_m1
Ccl5	Chemokine (C-C motif) ligand 5	Mm01302428_m1
Cd19	CD19 antigen	Mm00515420_m1
Cd3	CD3 antigen, epsilon polypeptide	Mm01179194_m1
Cd4	CD4 antigen	Mm00442754_m1
Cd68	CD68 antigen	Mm03047343_m1
Cd8a	CD8 antigen, alpha chain	Mm01182107_g1
Cdkn1b	Cyclin-dependent kinase inhibitor 1B	Mm00438167_g1
Cxcl10	Chemokine (C-X-C motif) ligand 10	Mm99999072_m1
Foxp3	Forkhead box P3	Mm00475162_m1
Gbp6	Guanylate binding protein 6	Mm00843395_m1
Gpr15	G protein-coupled receptor 15	Mm03990531_s1
Gpr15l (2610528A11Rik)	G protein-coupled receptor 15 ligand	Mm01213298_m1
Ifnβ	Interferon beta 1, fibroblast	Mm00439546_s1
Ifnγ	Interferon gamma	Mm00801778_m1
Il10	Interleukin 10	Mm00439616_m1
Irgm1	Immunity-related GTPase family M member 1	Mm00492596_m1
Parp14	Poly (ADP-ribose) polymerase family, member 14	Mm00520984_m1

Supplementary Table 2: Components of the CVB3 gene expression assay for random cDNA.

Assay	Gene name	Company	Final conc.
**CVB3_SE**	5'-CCCTGAATGCGGCTAATCC-3'	Invitrogen	2.5 µM
**CVB3_AS**	5'-ATTGTCACCATAAGCAGCCA-3'	Invitrogen	2.5 µM
**CVB3_FAM_probe**	5'-6-FAM-TGCAGCGGAACCG-MGB-3'	AppliedBiosystems	0.25 µM

6-FAM: 6-Carboxyfluorescein; MGB: *minor groove binder*,

Supplementary Table 3: Components of the CVB3 gene expression assay for strand-specific cDNA.

Assay	Gene name	Company	Final conc.
**CVB3_SE**	5’-TGAGATAATTGCCCTGAATGCG-3‘	Tib MolBiol	2.5 µM
**CVB3_AS**	5‘-CGCTTGATAGATTGTCACCATAAG-3‘	Tib MolBiol	2.5 µM
**CVB3_FAM_probe**	5‘-6-FAM-TGCAGCGGAACCG-MGB-3‘	AppliedBiosystems	0.25 µM

6-FAM: 6-Carboxyfluorescein; MGB: *minor groove binder.*

1.2 Chromogenic Immunohistochemistry

Supplementary Table 4: Antibodies used for chromogenic immunohistochemistry.

Target	Species	Dilution	Company	Order number	Clone
CD3	Rabbit	1:50	Abcam	Ab16669	SP7
CD8	Rabbit	1:500	Synaptic Systems	HS-361003	-
Histofine Simple Stain MAX PO (R), anti-rabbit, HRP	Goat	undiluted	medac-diagnostika	414141F	-

1.3 RNAscope *in situ* hybrisation

Supplementary Table 5: Antibodies, probes, and dyes used for RNAscope *in situ* hybridisation with subsequent immunohistological fluorescent staining.

Antibody/Reagent/Probe	Species	Dilution	Company	Order number
Mouse anti-TroponinT	Mouse	1:1500	Dianova	DLN-008802
Donkey anti mouse AlexaFluor488	Donkey	1:500	Invitrogen	A21202
Wheat germ agglutinin AlexaFluor633		1:500	Invitrogen	W21404
DAPI Fluoromount-G		undiluted	SouthernBiotech	0100-20
RNAscope® Probe-V-CVB3		undiluted	ACD	409291
RNAscope® Positive Control Probe-Mm-UBC		undiluted	ACD	310771
RNAscope® Negative Control Probe-DapB		undiluted	ACD	310043

1.4 Chemotaxis-assay and analysis of GPR15 expressing T cells

Supplementary Table 6: Primary antibodies with conjugated fluorophores used for FACS / flow cytometry analyses.

Antibody/Fluorophore	Species	Final concentration	Company	Order number	Clone
CD4_PE	Rat	2 ng/µl	eBioscience	12-0042-83	RM4-5
CD8_APC	Rat	2 ng/µl	BioLegend	100712	53-6.7
CD45_AlexaFluor700	Rat	2 ng/µl	eBioscience	56-0451-82	30-F11
CD25_PacificBlue	Rat	2 ng/µl	BioLegend	102021	PC61
Viability_PacificOrange	Rat	4 ng/µl	ThermoFisher	P30253	-

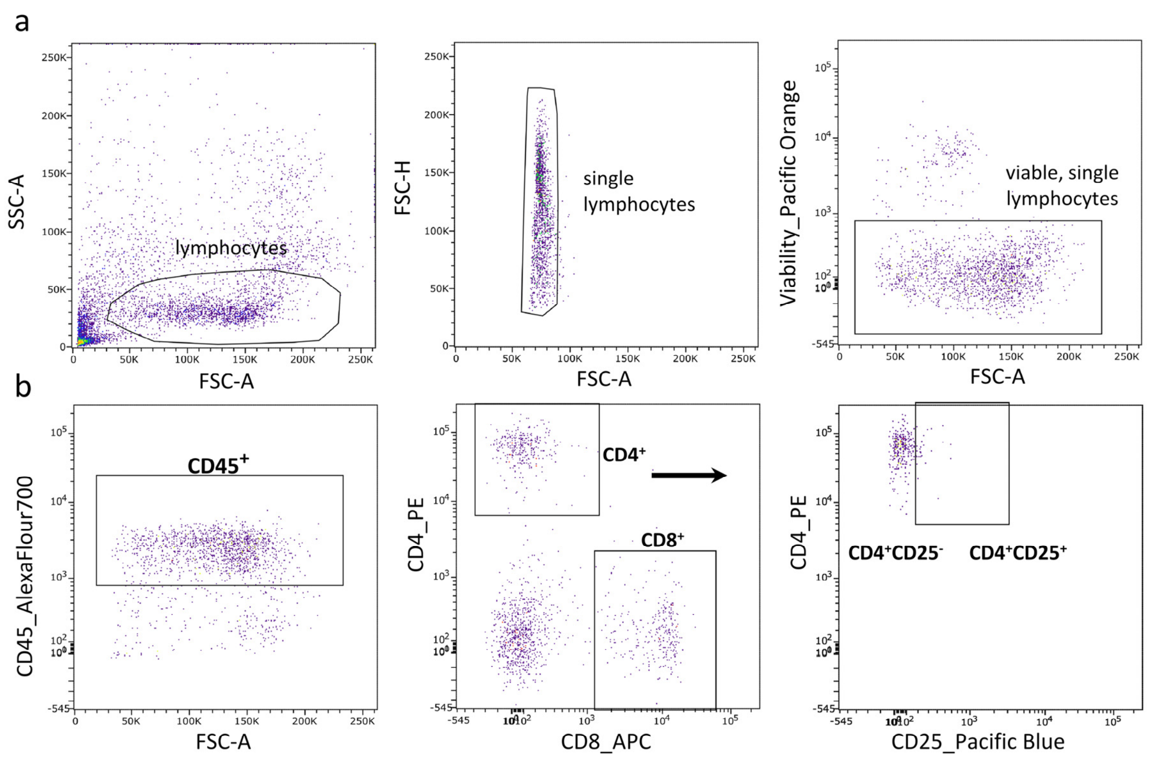

Supplementary Figure 1: Gating strategy to analyse chemotaxis experiments. Splenocytes (that had migrated through the pores of the inserts) were analysed via flow cytometry and gated as follows: (a) Lymphocytes were gated via forward (FSC) and sideward scatter (SSC) and then duplicates were excluded. Dead lymphocytes were excluded using Pacific Orange. (b) Based on the obtained cell population, the following immune cell subtypes were identified with primary fluorescence-labelled antibodies: $CD45^+$ lymphocytes, $CD8^+$ T_C, $CD4^+CD25^-$ T_H and $CD4^+CD25^+$ T_{reg} cells.

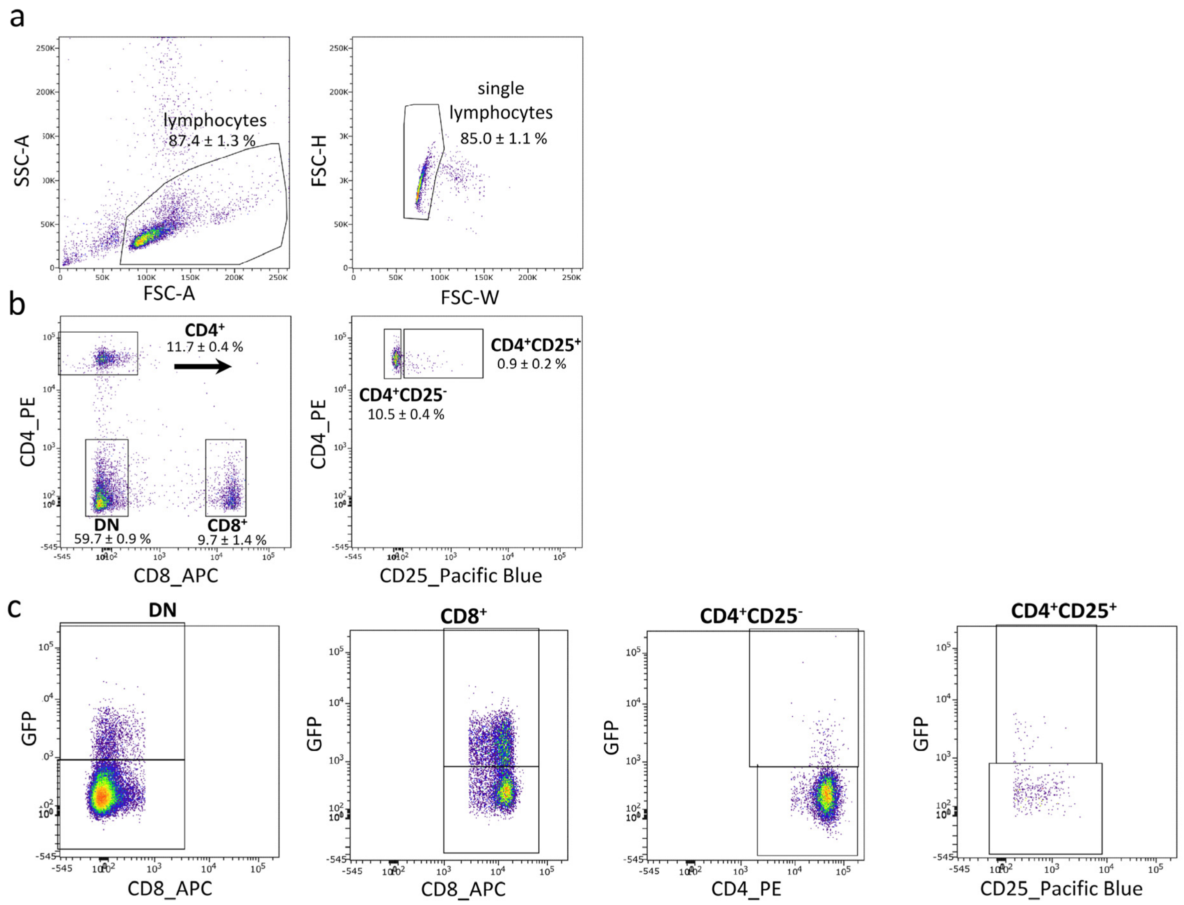

Supplementary Figure 2: Gating strategy to sort splenocytes or to quantify GFP$^+$ cells. Splenocytes were sorted via FACS or analysed via flow cytometry as follows: (a) Lymphocytes were gated via forward (FSC) and sideward scatter (SSC) and duplicates were excluded. (b) Based on the obtained cell population of single lymphocytes, the following immune cell subtypes were sorted with primary fluorescence-labelled antibodies: double negative (CD4$^-$CD8$^-$, DN). lymphocytes, CD8$^+$ T_C, CD4$^+$CD25$^-$ T_H and CD4$^+$CD25$^+$ T_{reg} cells. The percentage of the gated population is given as mean ± standard deviation. (c) Based on the gating strategy from A and B, GFP$^+$ cells were counted in the four groups: DN, CD8$^+$, CD4$^+$CD25$^-$ and CD4$^+$CD25$^+$ cells.

1.5 Actin polymerisation assay

Supplementary Table 7: Primary antibodies with conjugated fluorophores used for extracellular staining after phalloidin assay.

Antibody/Fluorophore	Species	Final concentration	Company	Order number	Clone
Phalloidin-iFluor 488		1x	Abcam	ab176753	
CD11b_PE-Cy7	Rat	0.4 ng/µl	Biolegend	101216	M1/70
CD45_PerCP	Rat	0.4 ng/µl	BioLegend	103132	30-F11
TCRb_ APC-eF780 or	Rat	0.4 ng/µl	eBioscience	47-5961-82	H57-597
CD3_APC	Rat	0.4 ng/µl	BioLegend	100236	17A2
CD4_APC or	Rat	0.6 ng/µl	BioLegend	100516	RM4-5
CD4_PerCP-Cy5.5	Rat	0.6 ng/µl	BioLegend	100431	GK1.5
CD8a_BV421	Rat	0.4 ng/µl	BioLegend	100753	53-6.7
CD25_PE	Rat	1 ng/µl	Invitrogen	12-0251-82	PC61.5
Viability_eFluor506		0.1%	eBioscience	65-0866-14	

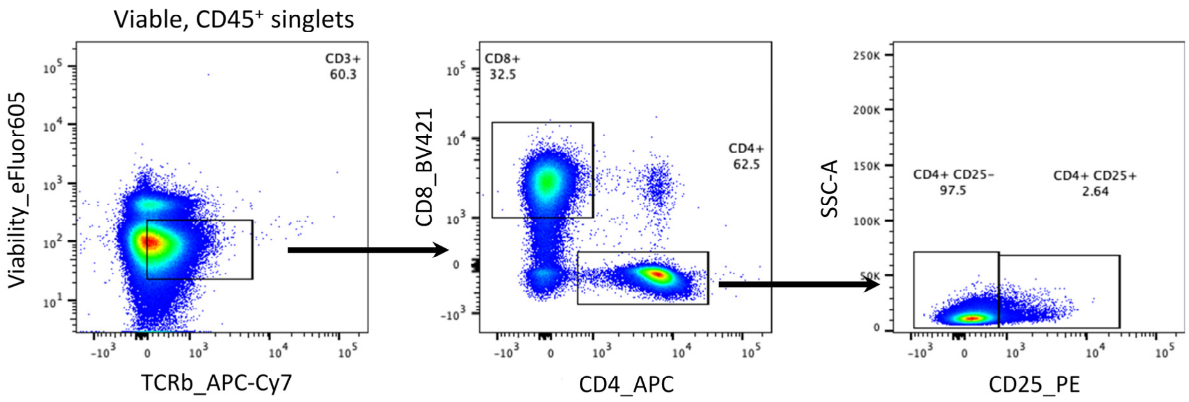

Supplementary Figure 3: Gating strategy to gate T cell subsets after phalloidin assay. Viable T cells were separeated in $CD8^+$ and $CD4^+$ cells, which were further subdivided in $CD4^+CD25^+$ and $CD4^+CD25^-$ cells.

1.6 IFNγ secretion assay

Supplementary Table 8: Primary antibodies with conjugated fluorophores used for interferon secretion assay.

Antibody/Fluorophore	Species	Final concentration	Company	Order number	Clone
CD8_PerCP-Cy5.5	Rat	1 ng/µl	BioLegend	100734	53-6.7
CD3_FITC	Rat	2.5 ng/µl	BioLegend	100204	17A2
CD25_APC	Rat	1 ng/µl	BioLegend	102012	PC61
CD45_PE-Cy7	Rat	1 ng/µl	BD	552848	30-F11
CD4_BV421	Rat	1 ng/µl	BioLegend	100443	GK1.5
Viability_eFluor506		0.1%	eBioscience	65-0866-14	

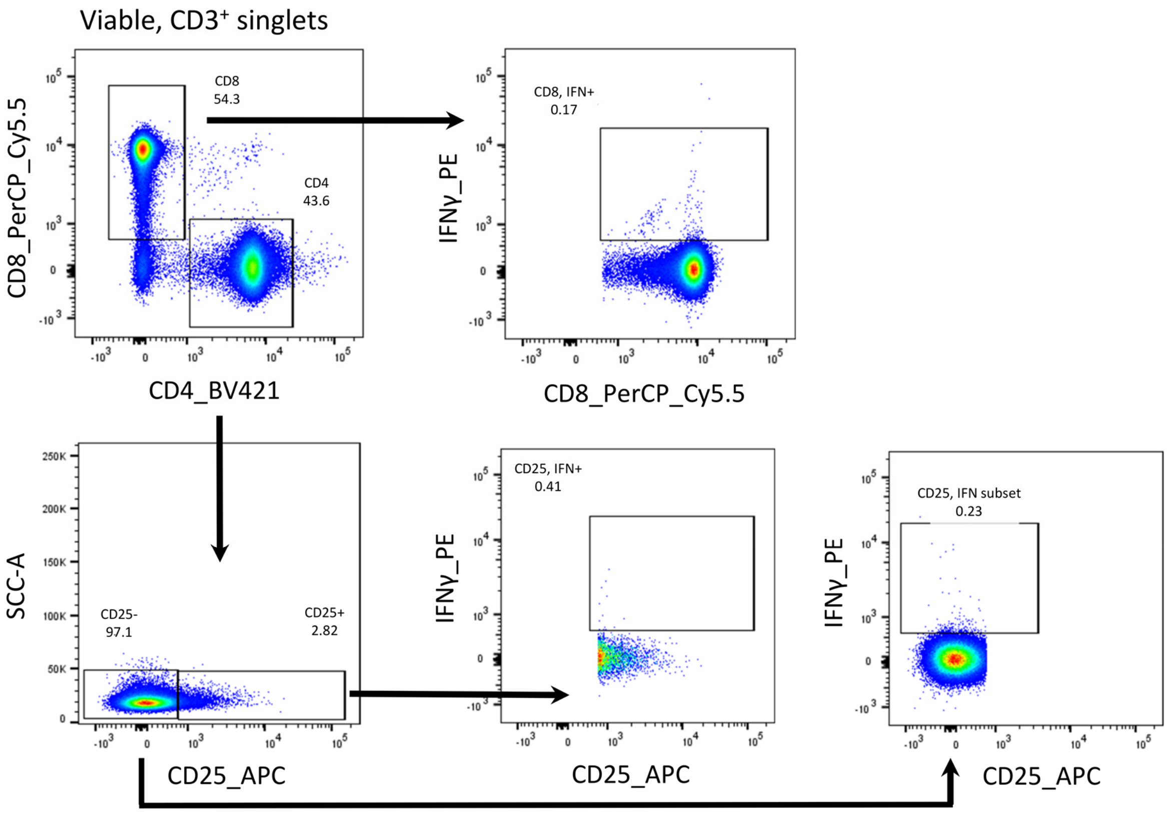

Supplementary Figure 4: Gating strategy to gate T cell subsets after IFNγ secretion assay. Viable CD3+ T cell singlets were separeated in CD8+ and CD4+ cells, which were further subdivided in CD4+CD25+ and CD4+CD25- cells. For each T cell subsets, proportion of IFNγ+ cells was determined.

1.7 T cell activation assay

Supplementary Table 9: Primary antibodies with conjugated fluorophores used for extracellular staining.

Antibody/Fluorophore	Species	Final concentration	Company	Order number	Clone
CD45_PerCP	Rat	2 ng/µl	BioLegend	103132	30-F11
CD11b_AF700	Rat	5 ng/µl	BioLegend	101222	M1/70
CD11c_AF700	Armenian hamster	5 ng/µl	BioLegend	117320	N418
CD19_AF700	Rat	5 ng/µl	BioLegend	115528	6D5
F4/80_AF700	Rat	5 ng/µl	BioLegend	123130	BM8
Ly6G_AF700	Rat	5 ng/µl	BioLegend	127622	1A8
TER-119_AF700	Rat	5 ng/µl	BioLegend	116220	TER-119
CD3_APC	Rat	2 ng/µl	BioLegend	100236	17A2
FR4_PE-Cy7	Rat	2 ng/µl	BioLegend	125012	12A5
CD4_PE/Dazzle	Rat	2 ng/µl	BioLegend	100566	RM4-5
CD25_PE	Rat	4 ng/µl	eBioscience	12-0251-82	PC61.5
CD8a_BV421	Rat	2 ng/µl	BioLegend	100753	53-6.7
Viability_APC-Cy7		0.1%	eBioscience	65-0865-18	-

Supplementary Table 10: Primary antibodies with conjugated fluorophores used for intracellular staining.

Antibody/Fluorophore	Species	Final concentration	Company	Order number	Clone
GranzB_FITC	Mouse	4 ng/µl	BioLegend	372206	QA16A02
IL-17_BV785	Rat	4 ng/µl	BioLegend	506928	TC11-18H10.1
IFNγ_BV711	Rat	4 ng/µl	BioLegend	505836	XMG1.2
TNFα_BV650	Rat	4 ng/µl	BioLegend	506333	MP6-XT22
IL-10_BV605	Rat	4 ng/µl	BioLegend	505031	JES5-16E3

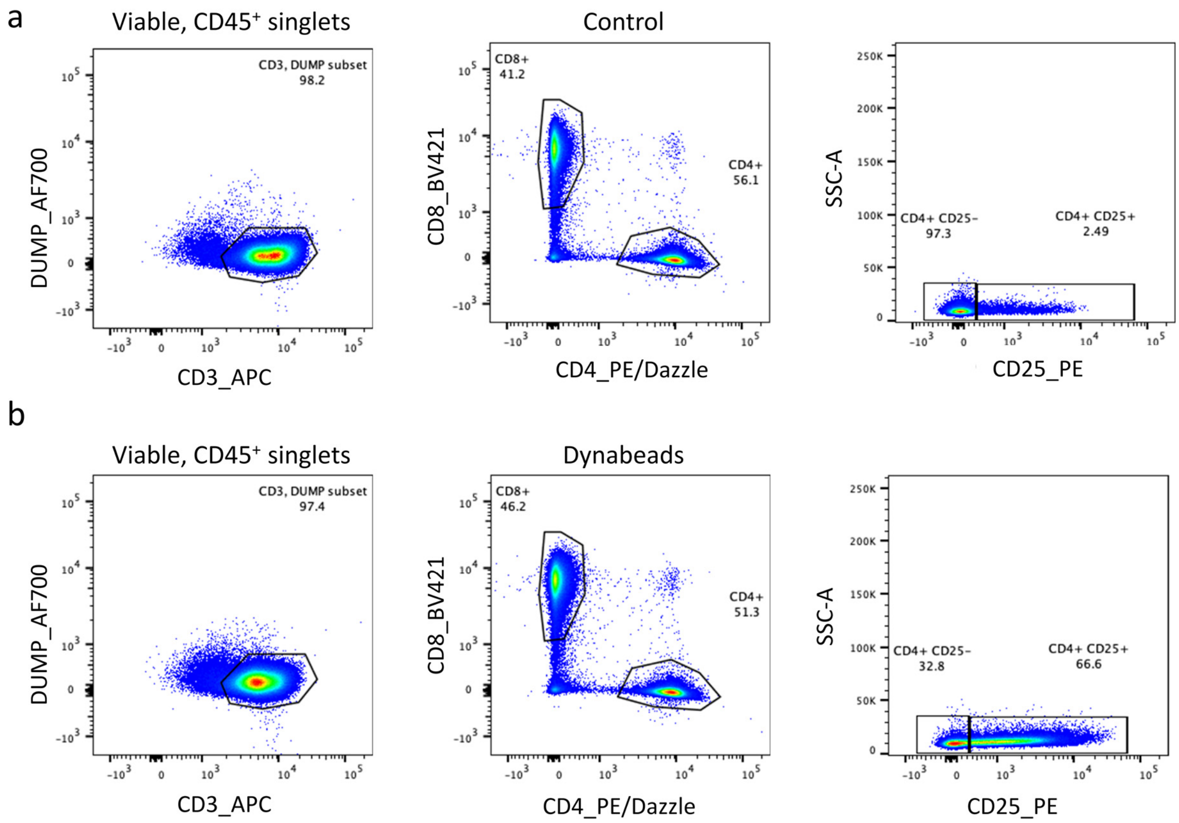

Supplementary Figure 5: Gating strategy to gate T cell subsets after stimulation with Dynabeads and GPR15L. Viable T cells were separated in $CD8^+$ and $CD4^+$ cells, which were further subdivided in $CD4^+CD25^+$ and $CD4^+CD25^-$ cells.
