# Supplementary material for: GPR15-mediated T cell recruitment during acute viral myocarditis facilitated virus elimination and improved outcome
Source: Nat Cardiovasc Res. 2023 Dec 27;3(1):76–93. doi: 10.1038/s44161-023-00401-z (PMC11357984; doi:10.1038/s44161-023-00401-z)
Supplement: Supplementary file 2 — Reporting Summary [file 44161_2023_401_MOESM2_ESM.pdf]

nature portfolio

Corresponding author(s): Diana Lindner

Last updated by author(s): Nov 22, 2023

Reporting Summary

Nature Portfolio wishes to improve the reproducibility of the work that we publish. This form provides structure for consistency and transparency in reporting. For further information on Nature Portfolio policies, see our Editorial Policies and the Editorial Policy Checklist.

Statistics

For all statistical analyses, confirm that the following items are present in the figure legend, table legend, main text, or Methods section.

n/a	Confirmed	
☐	☒	The exact sample size (*n*) for each experimental group/condition, given as a discrete number and unit of measurement
☐	☒	A statement on whether measurements were taken from distinct samples or whether the same sample was measured repeatedly
☐	☒	The statistical test(s) used AND whether they are one- or two-sided<br>*Only common tests should be described solely by name; describe more complex techniques in the Methods section.*
☒	☐	A description of all covariates tested
☐	☒	A description of any assumptions or corrections, such as tests of normality and adjustment for multiple comparisons
☐	☒	A full description of the statistical parameters including central tendency (e.g. means) or other basic estimates (e.g. regression coefficient) AND variation (e.g. standard deviation) or associated estimates of uncertainty (e.g. confidence intervals)
☐	☒	For null hypothesis testing, the test statistic (e.g. *F*, *t*, *r*) with confidence intervals, effect sizes, degrees of freedom and *P* value noted<br>*Give P values as exact values whenever suitable.*
☒	☐	For Bayesian analysis, information on the choice of priors and Markov chain Monte Carlo settings
☒	☐	For hierarchical and complex designs, identification of the appropriate level for tests and full reporting of outcomes
☒	☐	Estimates of effect sizes (e.g. Cohen's *d*, Pearson's *r*), indicating how they were calculated

Our web collection on statistics for biologists contains articles on many of the points above.

Software and code

Policy information about availability of computer code

Data collection	BZ II Analyzer software, FACSDiva software (version 9.0.1), iox2 software 2.9.5.73
Data analysis	LabChart 7.3Pro, QuantStudioTM software v1.3, DESeq2 v1.20, Cutadapt V2.3, ComplexHeatmap 3.6.3, Graph Pad Prism 6.07 and 9.5.1, topGO version 2.42.0, simplifyEnrichment 1.0.0, ggforce version 0.3.3, ggplot2 version 3.3.3, GOplot version 1.0.2, QuPath version 0.4.3, FCSalyzer software Version 0.9.22-alpha, FlowJo version 10.8.1, FIJI software (2.14.0), R studio Version 4.0.2, G*Power Version 3.1.9.7, Design and Analysis software 2.6.0, Leica LAS AF software

For manuscripts utilizing custom algorithms or software that are central to the research but not yet described in published literature, software must be made available to editors and reviewers. We strongly encourage code deposition in a community repository (e.g. GitHub). See the Nature Portfolio guidelines for submitting code & software for further information.

Data

Policy information about availability of data

All manuscripts must include a data availability statement. This statement should provide the following information, where applicable:

- Accession codes, unique identifiers, or web links for publicly available datasets
- A description of any restrictions on data availability
- For clinical datasets or third party data, please ensure that the statement adheres to our policy

mouse genome (mm10)

All data generated during and/or analysed during the current study are included in the main article and associated files or are available from the corresponding author on reasonable request.

Research involving human participants, their data, or biological material

Policy information about studies with human participants or human data. See also policy information about sex, gender (identity/presentation), and sexual orientation and race, ethnicity and racism.

Reporting on sex and gender	N/A
Reporting on race, ethnicity, or other socially relevant groupings	N/A
Population characteristics	N/A
Recruitment	N/A
Ethics oversight	N/A

Note that full information on the approval of the study protocol must also be provided in the manuscript.

Field-specific reporting

Please select the one below that is the best fit for your research. If you are not sure, read the appropriate sections before making your selection.

☒ Life sciences ☐ Behavioural & social sciences ☐ Ecological, evolutionary & environmental sciences

For a reference copy of the document with all sections, see nature.com/documents/nr-reporting-summary-flat.pdf

Life sciences study design

All studies must disclose on these points even when the disclosure is negative.

Sample size	Our published data were used to determine the variability and expected changes of the study and those values were used to calculate the sample size by G*Power Version 3.1.9.7
Data exclusions	Mice were excluded from the analyses, If they did not exhibit virus load in blood at day 4 p.i. and in LV tissue later. they were and was therefore excluded from the study. Mice with too deep anaesthesia or excessive bleeding during the haemodynamic measurement were excluded from the analysis. Exclusions were clearly described throughout the manuscript. For chromogenic immunhistological staining and subsequent quantification of CD3+ and CD8+ T cells in LV tissue, for 1 GPR15-deficient CVB3 infected mouse (5d) FFPE tissue was not collected and therefore not available. Furthermore, for 1 GPR15-deficient CVB3 infected mouse (5d) and 1 WT CVB3 infected mouse (5d) CD8 staining was not successfull.
Replication	Reproduciblilitv of experimental findings was corroborated in independent experiments. We were able to produce similar results in the independent mouse experiments. The individual captions in the main text indicate the number of times each experiment was replicated or performed independently.
Randomization	KO and WT mice were randomly subjected to either CVB3 or NaCl injection. Whenever possible isolated cells or cell lines where pooled prior to following treatment to avoid changes during isolation or proliferation.
Blinding	All investigators were blinded to the group or genotype of analyzed tissue. During heart function measurments infected mice were clearly discriminable from healthy mice, however genotype was blinded. For in vitro studies, investigators were unblinded since the same investigators both conducted and analyzed the experiments. Blinding was not relevant for FACS experiments because the same gating strategies were used for all samples during analysis.

Reporting for specific materials, systems and methods

We require information from authors about some types of materials, experimental systems and methods used in many studies. Here, indicate whether each material, system or method listed is relevant to your study. If you are not sure if a list item applies to your research, read the appropriate section before selecting a response.

Materials & experimental systems

n/a	Involved in the study
☐	☒ Antibodies
☐	☒ Eukaryotic cell lines
☒	☐ Palaeontology and archaeology
☐	☒ Animals and other organisms
☒	☐ Clinical data
☒	☐ Dual use research of concern
☒	☐ Plants

Methods

n/a	Involved in the study
☒	☐ ChIP-seq
☐	☒ Flow cytometry
☒	☐ MRI-based neuroimaging

Antibodies

Antibodies used

For FcγR blocking, 500 ng/ml anti-CD16/CD32 monoclonal antibody was used (clone 2.4G2, BioXcell, BE0008).
All other antibodies (clones, sources and dilutions) are described in the supplemental tables S4, S5, S6, S7, S8, S9 and S10 of the paper.

Validation

All antibodies used in the manuscript have been validated commercially and in our hands using appropriate positive and negative controls.
anti-CD16/CD32 monoclonal antibody (clone 2.4G2, BioXcell, BE0008); Trefzer A et al. 2021, Cell Reports, PMID:108748; Pezoldt J et al. Nat Commun 13, 7227 (2022). https://doi.org/10.1038/s41467-022-34868-4
CD3 (ab16669): https://www.abcam.com/products/primary-antibodies/cd3-epsilon-antibody-sp7-ab16669.html
CD8 (HS-361003): https://sysy.com/product/HS-361003
Donkey anti mouse AlexaFluor488 (A21202): https://www.thermofisher.com/order/genome-database/dataSheetPdf?producttype=antibody&productsubtype=antibody_secondary&productId=A-21202&version=359
Wheat germ agglutinin AlexaFluor633 (W21404): https://www.thermofisher.com/order/catalog/product/de/de/W21404
CD4_PE (12-0042-83): https://www.thermofisher.com/order/genome-database/dataSheetPdf?producttype=antibody&productsubtype=antibody_primary&productId=12-0042-82&version=359
CD8_APC (100712): https://www.biolegend.com/en-us/products/apc-anti-mouse-cd8a-antibody-150?pdf=true&displayInline=true&leftRightMargin=15&topBottomMargin=15&filename=APC%20anti-mouse%20CD8a%20Antibody.pdf&v=20230714033116
CD45_AlexaFluor700 (56-0451-82): https://www.thermofisher.com/order/genome-database/dataSheetPdf?producttype=antibody&productsubtype=antibody_primary&productId=56-0451-82&version=359
CD25_PacificBlue (102021): https://d1spbj2x7qk4bg.cloudfront.net/en-ie/products/pacific-blue-anti-mouse-cd25-antibody-3315?pdf=true&displayInline=true&leftRightMargin=15&topBottomMargin=15&filename=Pacific%20Blue%E2%84%A2%20anti-mouse%20CD25%20Antibody.pdf&v=20230714033116
Viability_PacificOrange (P30253): https://www.thermofisher.com/order/catalog/product/de/de/P30253
Phalloidin-iFluor 488 (ab176753): https://www.abcam.com/products/chip-kits/phalloidin-ifluor-488-reagent-ab176753.html
CD11b_PE-Cy7 (101216): https://www.biolegend.com/de-de/cell-health/pe-cyanine7-anti-mouse-human-cd11b-antibody-1921?pdf=true&displayInline=true&leftRightMargin=15&topBottomMargin=15&filename=PE/Cyanine7%20anti-mouse/human%20CD11b%20Antibody.pdf&v=20230726063409
CD45_PerCP (103132): https://d1spbj2x7qk4bg.cloudfront.net/en-gb/products/percp-cyanine5-5-anti-mouse-cd45-antibody-4264?pdf=true&displayInline=true&leftRightMargin=15&topBottomMargin=15&filename=PerCP/Cyanine5.5%20anti-mouse%20CD45%20Antibody.pdf&v=20230114013553
TCRb_APC-eFluor™ 780(47-5961-82): https://www.thermofisher.com/order/genome-database/dataSheetPdf?producttype=antibody&productsubtype=antibody_primary&productId=47-5961-82&version=359
CD3_APC (100236): https://d1spbj2x7qk4bg.cloudfront.net/fr-ch/products/apc-anti-mouse-cd3-antibody-8055?pdf=true&displayInline=true&leftRightMargin=15&topBottomMargin=15&filename=APC%20anti-mouse%20CD3%20Antibody.pdf&v=20230726063409
CD4_APC (100516): https://d1spbj2x7qk4bg.cloudfront.net/ja-jp/products/apc-anti-mouse-cd4-antibody-477?pdf=true&displayInline=true&leftRightMargin=15&topBottomMargin=15&filename=APC%20anti-mouse%20CD4%20Antibody.pdf&v=20230726063409
CD4_PerCP-Cy5.5 (100431): https://d1spbj2x7qk4bg.cloudfront.net/ja-jp/products/percp-anti-mouse-cd4-antibody-4219?pdf=true&displayInline=true&leftRightMargin=15&topBottomMargin=15&filename=PerCP%20anti-mouse%20CD4%20Antibody.pdf&v=20220421053143
CD8a_BV421 (100753): https://d1spbj2x7qk4bg.cloudfront.net/nl-nl/products/brilliant-violet-421-anti-mouse-cd8a-antibody-7138?pdf=true&displayInline=true&leftRightMargin=15&topBottomMargin=15&filename=Brilliant%20Violet%20421%E2%84%A2%20anti-mouse%20CD8a%20Antibody.pdf&v=20230920123134
CD25_PE (12-0251-82): https://www.thermofisher.com/order/genome-database/dataSheetPdf?producttype=antibody&productsubtype=antibody_primary&productId=12-0251-82&version=359
Viability_eFluor506 (65-0866-14): https://www.thermofisher.com/order/catalog/product/de/de/65-0866-14
CD8_PerCP-Cy5.5 (100734): https://d1spbj2x7qk4bg.cloudfront.net/en-ie/products/percp-cyanine5-5-anti-mouse-cd8a-antibody-4255?pdf=true&displayInline=true&leftRightMargin=15&topBottomMargin=15&filename=PerCP/Cyanine5.5%20anti-mouse%20CD8a%20Antibody.pdf&v=20230714033116
CD3_FITC (100204): https://www.biolegend.com/de-de/explore-new-products/fitc-anti-mouse-cd3-antibody-45?GroupID=BLG6732
CD25_APC (102012): https://d1spbj2x7qk4bg.cloudfront.net/fr-lu/products/apc-anti-mouse-cd25-antibody-420?pdf=true&displayInline=true&leftRightMargin=15&topBottomMargin=15&filename=APC%20anti-mouse%20CD25%20Antibody.pdf&v=20231114073227
CD45_PE-Cy7 (552848): https://www.bdbiosciences.com/en-us/products/reagents/flow-cytometry-reagents/research-reagents/single-color-antibodies-ruo/pe-cy-7-rat-anti-mouse-cd45.552848
CD4_BV421 (100443): https://www.biolegend.com/en-us/search-results/brilliant-violet-421-anti-mouse-cd4-antibody-7142?

pdf=true&displayInline=true&leftRightMargin=15&topBottomMargin=15&filename=Brilliant%20Violet%20421%E2%84%A2%20anti-mouse%20CD4%20Antibody.pdf&v=20230803063053
CD45_PerCP (103132): https://d1spbj2x7qk4bg.cloudfront.net/en-gb/products/percp-cyanine5-5-anti-mouse-cd45-antibody-4264?pdf=true&displayInline=true&leftRightMargin=15&topBottomMargin=15&filename=PerCP/Cyanine5.5%20anti-mouse%20CD45%20Antibody.pdf&v=20230114013553
CD11b_AF700 (101222): https://www.biolegend.com/en-us/search-results/alexa-fluor-700-anti-mouse-human-cd11b-antibody-3388?pdf=true&displayInline=true&leftRightMargin=15&topBottomMargin=15&filename=Alexa%20Fluor%C2%AE%20700%20anti-mouse/human%20CD11b%20Antibody.pdf&v=20230726063409
CD11c_AF700 (117320): https://d1spbj2x7qk4bg.cloudfront.net/fr-lu/products/alexa-fluor-700-anti-mouse-cd11c-antibody-3429?pdf=true&displayInline=true&leftRightMargin=15&topBottomMargin=15&filename=Alexa%20Fluor%C2%AE%20700%20anti-mouse%20CD11c%20Antibody.pdf&v=20231114073227
CD19_AF700 (115528): https://www.biolegend.com/de-de/cell-health/alexa-fluor-700-anti-mouse-cd19-antibody-3391?pdf=true&displayInline=true&leftRightMargin=15&topBottomMargin=15&filename=Alexa%20Fluor%C2%AE%20700%20anti-mouse%20CD19%20Antibody.pdf&v=20230714033116
F4/80_AF700 (123130): https://d1spbj2x7qk4bg.cloudfront.net/en-gb/products/alexa-fluor-700-anti-mouse-f4-80-antibody-6556?pdf=true&displayInline=true&leftRightMargin=15&topBottomMargin=15&filename=Alexa%20Fluor%C2%AE%20700%20anti-mouse%20F4/80%20Antibody.pdf&v=20230114013553
Ly6G_AF700 (127622): https://d1spbj2x7qk4bg.cloudfront.net/fr-lu/products/alexa-fluor-700-anti-mouse-ly-6g-antibody-6754?pdf=true&displayInline=true&leftRightMargin=15&topBottomMargin=15&filename=Alexa%20Fluor%C2%AE%20700%20anti-mouse%20Ly-6G%20Antibody.pdf&v=20230801063041
TER-119_AF700 (116220): https://d1spbj2x7qk4bg.cloudfront.net/de-de/products/alexa-fluor-700-anti-mouse-ter-119-erythroid-cells-antibody-3428?pdf=true&displayInline=true&leftRightMargin=15&topBottomMargin=15&filename=Alexa%20Fluor%C2%AE%20700%20anti-mouse%20TER-119/Erythroid%20Cells%20Antibody.pdf&v=20230701123045
FR4_PE-Cy7 (125012): https://d1spbj2x7qk4bg.cloudfront.net/de-de/products/pe-cyanine7-anti-mouse-fr4-folate-receptor-4-antibody-4924?pdf=true&displayInline=true&leftRightMargin=15&topBottomMargin=15&filename=PE/Cyanine7%20anti-mouse%20FR4%20(Folate%20Receptor%204)%20Antibody.pdf&v=20231114073227
CD4_PE/Dazzle (100566): https://d1spbj2x7qk4bg.cloudfront.net/de-de/products/pe-dazzle-594-anti-mouse-cd4-antibody-9845?pdf=true&displayInline=true&leftRightMargin=15&topBottomMargin=15&filename=PE/Dazzle%E2%84%A2%20594%20anti-mouse%20CD4%20Antibody.pdf&v=20230223043110
Viability_APC-Cy7 (65-0865-18): https://www.thermofisher.com/order/catalog/product/de/de/65-0865-18
GranzB_FITC (372206): https://d1spbj2x7qk4bg.cloudfront.net/en-gb/products/fitc-anti-human-mouse-granzyme-b-recombinant-antibody-14430?pdf=true&displayInline=true&leftRightMargin=15&topBottomMargin=15&filename=FITC%20anti-human/mouse%20Granzyme%20B%20Recombinant%20Antibody.pdf&v=20221115073101
IL-17_BV785 (506928): https://d1spbj2x7qk4bg.cloudfront.net/de-de/products/brilliant-violet-785-anti-mouse-il-17a-antibody-7988?pdf=true&displayInline=true&leftRightMargin=15&topBottomMargin=15&filename=Brilliant%20Violet%20785%E2%84%A2%20anti-mouse%20IL-17A%20Antibody.pdf&v=20230114013553
IFNγ_BV711 (505836): https://d1spbj2x7qk4bg.cloudfront.net/en-gb/search-results/brilliant-violet-711-anti-mouse-ifn-gamma-antibody-7950?pdf=true&displayInline=true&leftRightMargin=15&topBottomMargin=15&filename=Brilliant%20Violet%20711%E2%84%A2%20anti-mouse%20IFN-%CE%B3%20Antibody.pdf&v=20230726063409
TNFα_BV650 (506333): https://d1spbj2x7qk4bg.cloudfront.net/fr-lu/products/brilliant-violet-650-anti-mouse-tnf-alpha-antibody-8829?pdf=true&displayInline=true&leftRightMargin=15&topBottomMargin=15&filename=Brilliant%20Violet%20650%E2%84%A2%20anti-mouse%20TNF-%CE%B1%20Antibody.pdf&v=20230803063053
IL-10_BV605 (505031): https://d1spbj2x7qk4bg.cloudfront.net/en-gb/products/brilliant-violet-605-anti-mouse-il-10-antibody-9382?pdf=true&displayInline=true&leftRightMargin=15&topBottomMargin=15&filename=Brilliant%20Violet%20605%E2%84%A2%20anti-mouse%20IL-10%20Antibody.pdf&v=20230105073058

Eukaryotic cell lines

Policy information about cell lines and Sex and Gender in Research

Cell line source(s)	MHEC5-T were purched from DSMZ (ACC 336).<br>HL1 were purched from Merck (SCC065).
Authentication	Cell lines were purchased from DSMZ (MHEC5-T) and Merck (HL1). Morphological assessment via microscopy was used to ensure origin of cell line.
Mycoplasma contamination	no mycoplasma contamination tested.
Commonly misidentified lines<br>(See ICLAC register)	Commonly misidentified cell lines were not used in this study.

Animals and other research organisms

Policy information about studies involving animals; ARRIVE guidelines recommended for reporting animal research, and Sex and Gender in Research

Laboratory animals	We used C57BL6 WT mice and the knock-in mouse strain (B6; 129P2-Gpr15tm1.1Litt/J) at the age of 7 to 10 weeks to induce viral myocarditis in our studies. Primary murine cardiac fibroblasts were obtained from male WT B6 mice at the age of 10-12 weeks. Primary lymphocytes from spleen were isolated from male WT and GPR15-deficient B6 mice at the age of 8-16 weeks. All mice were kept under pathogen-free conditions in the laboratory animal facility University Hospital Hamburg-Eppendorf at 22°C with a 12 hour light/dark cycle and free access to water and standard laboratory chow.

Wild animals	This study did not include wild animals.
Reporting on sex	Male mice were exclusively used in this study.
Field-collected samples	This study did not involve samples collected from the field.
Ethics oversight	All animal experiments were approved by the local bioethics committee of Hamburg, Germany (G13/115, G15/060, N060/2020, ORG821, ORG1068) and conform to the "Guide for the Care and Use of Laboratory Animals" published by the US NRC (8th edition, revised 2011).

Note that full information on the approval of the study protocol must also be provided in the manuscript.

Plants

Seed stocks	N/A
Novel plant genotypes	N/A
Authentication	N/A

Flow Cytometry

Plots

Confirm that:

☒ The axis labels state the marker and fluorochrome used (e.g. CD4-FITC).

☒ The axis scales are clearly visible. Include numbers along axes only for bottom left plot of group (a 'group' is an analysis of identical markers).

☒ All plots are contour plots with outliers or pseudocolor plots.

☒ A numerical value for number of cells or percentage (with statistics) is provided.

Methodology

Sample preparation	Cells are freshly isolated from mice spleens and stained with the indicated antibodies prior to FACS analyses.
Instrument	Flow cytometry analysis was performed on LSR Fortessa™, FACSCanto and a FACSCanto II, BD. Sorting was performed on AriaIIIu, BD
Software	Data were collected using BD FACS diva software. Flow cytometry data were analysed using FCSalyzer or FlowJo software.
Cell population abundance	Abundance of T-cell subtypes is given for sorting experiments in the supplemental part.<br>Sorting of splenocytes: The percentages of the populations are: CD4-CD8-, DN: 59.7 ± 0.7 %; CD8+ TC: 9.7 ± 1.4 %; CD4 +CD25- TH: 10.5 ± 0.4 % and CD4+CD25+ Treg: 0.9 ± 0.2 %.<br>Sorting of T cells:<br>The percentages of the populations for stimulated cells are: CD4-CD8-, DN: 2.1 ± 0.7 of CD3+ cells; CD8+: 50.0 ± 5.3 of CD3+ cells; CD4+CD25- 27.7 ± 15.7 of CD4+ cells; CD4+CD25+ 60.7 ± 25.0 of CD4+ cells<br>The percentages of the for unstimulated cells populations are: CD4-CD8-, DN: 2.7 ± 0.2 of CD3+ cells; CD8+: 40.8 ± 4.4 of CD3 + cells; CD4+CD25- 95.3 ± 0.9 of CD4+ cells; CD4+CD25+ 2.6 ± 1.5 of CD4+ cells
Gating strategy	All gating strategies are defined in the supplemental part for each experiment.<br>Gating strategy to sort splenocytes or to quantify GFP+ cells: Lymphocytes were gated via FSC and SSC and duplicates were excluded. Based on the obtained cell population of single lymphocytes, the following immune cell subtypes were sorted with primary fluorescence-labelled antibodies: double negative (CD4-CD8-, DN). lymphocytes, CD8+ TC, CD4+CD25- TH and CD4 +CD25+ Treg cells.<br>Gating strategy to gate T cell subsets after phalloidin assay: Lymphocytes were gated via FSC and SSC and duplicates were excluded. Viable T cells were separated in CD8+ and CD4+ cells, which were further subdivided in CD4+CD25+ and CD4 +CD25- cells.<br>Gating strategy to gate T cell subsets after IFNγ secretion assay: Lymphocytes were gated via FSC and SSC and duplicates were excluded. Viable CD3+ T cell singlets were separeated in CD8+ and CD4+ cells, which were further subdivided in CD4 +CD25+ and CD4+CD25- cells. For each T cell subsets, proportion of IFNγ+ cells was determined<br>Gating strategy to gate T cell subsets after stimulation with Dynabeads and GPR15L: Lymphocytes were gated via FSC and SSC and duplicates were excluded. Viable T cells were separated in CD8+ and CD4+ cells, which were further subdivided in CD4

+CD25+ and CD4+CD25- cells.

☒ Tick this box to confirm that a figure exemplifying the gating strategy is provided in the Supplementary Information.
